# Self-stretching molecular monolayers with autonomous folding and mechanical adaptation

**DOI:** 10.1126/sciadv.aeg8906

**Published:** 2026-09-09

**Authors:** Taichi Iizuka, Satoru Inoue, Kiyoshi Nikaido, Seiji Tsuzuki, Saori Maki-Yonekura, Tasuku Hamaguchi, Koji Yonekura, Tatsuo Hasegawa

**Affiliations:** ^1^ Department of Applied Physics, The University of Tokyo, Tokyo 113-8656, Japan.; ^2^ Innovation Center for Organic Electronics (INOEL), Yamagata University, Yonezawa 992-0119, Japan.; ^3^ Biostructural Mechanism Laboratory, RIKEN SPring-8 Center, Kouto, Hyogo 679-5148, Japan.; ^4^ Institute of Multidisciplinary Research for Advanced Materials, Tohoku University, Katahira, Sendai 980-8577, Japan.

## Abstract

Molecular monolayers at fluid interfaces are generally considered mechanically fragile, collapsing under lateral compression. Here we show that a rationally designed amphiphilic π-conjugated molecule instead forms a monolayer that adapts to mechanical stress. Langmuir films of 3-hydroxyphenyl-7-decyl-[1]benzothieno[3,2-*b*][1]benzothiophene (HP-BTBT-C10) accommodate and store compressive stress without collapse. In situ optical imaging reveals that lateral compression converts into reversible out-of-plane deformation via anisotropic molecular reorganization, producing ordered wrinkles. Electron diffractometry and high-resolution electron microscopy show localized multilayer folding while retaining local molecular packing. Intermolecular interaction calculations suggest that hydroxy substitution helps stabilize both the Langmuir monolayer on water and folded multilayer wrinkles, while preserving the strong layer-forming tendency of alkyl-substituted extended π-core molecules. Dynamic measurements based on barrier-oscillation experiments further demonstrate repeatable viscoelastic stress responses mediated by sequential folding and wrinkle reversibility, establishing mechanically adaptive molecular monolayers that bridge conventional Langmuir films and biologically inspired membranes.

## INTRODUCTION

Biological membranes dynamically adapt to mechanical stimuli by converting in-plane stress—whether externally applied or internally generated—into out-of-plane deformation, thereby avoiding catastrophic failure while maintaining structural integrity (*1*, *2*). Such mechanically adaptive behavior, manifested as folding, protrusion formation, and stress dissipation through collective molecular rearrangement, underlies diverse biological functions. Reproducing this adaptive response in artificial molecular monolayers, however, has remained a longstanding challenge (*3*–*6*).

Conventional fatty-acid Langmuir monolayers at the air–water interface behave as mechanically fragile two-dimensional solids: lateral compression leads to densification followed by abrupt collapse, with little capacity for reversible stress accommodation (*7*–*10*). Although theoretical and experimental studies have examined instabilities such as buckling, wrinkling, and collapse precursors (*11*–*15*), these processes generally represent irreversible failure rather than adaptive mechanical response.

Recent advances in molecular design have highlighted the ability of π-conjugated systems to form highly ordered yet mechanically compliant assemblies through combined intermolecular interactions (*16*–*25*). Nevertheless, even in such systems, interfacial monolayers are typically regarded as quasi-static structures whose mechanical response is limited to elastic compression or irreversible collapse. Whether a rationally designed molecular monolayer can adapt to mechanical stress by dynamically reorganizing while retaining molecular order has remained unresolved.

Here we show that an amphiphilic π-conjugated molecule, HP-BTBT-C10, forms a Langmuir monolayer exhibiting a distinct mode of mechanical adaptation. Under lateral compression, HP-BTBT-C10 monolayers accommodate in-plane stress through continuous internal rearrangement rather than collapse, resulting in persistent stress storage and reversible deformation. Using in situ optical imaging, electron diffractometry, high-resolution electron microscopy, and dynamic mechanical measurements, we demonstrate that this adaptive behavior arises from reversible conversion of in-plane compression into out-of-plane deformation mediated by localized multilayer folding while retaining local molecular order, culminating in ordered wrinkle formation.

## RESULTS

### Langmuir compression of HP-BTBT-C10 monolayers

**Fig. 1.**
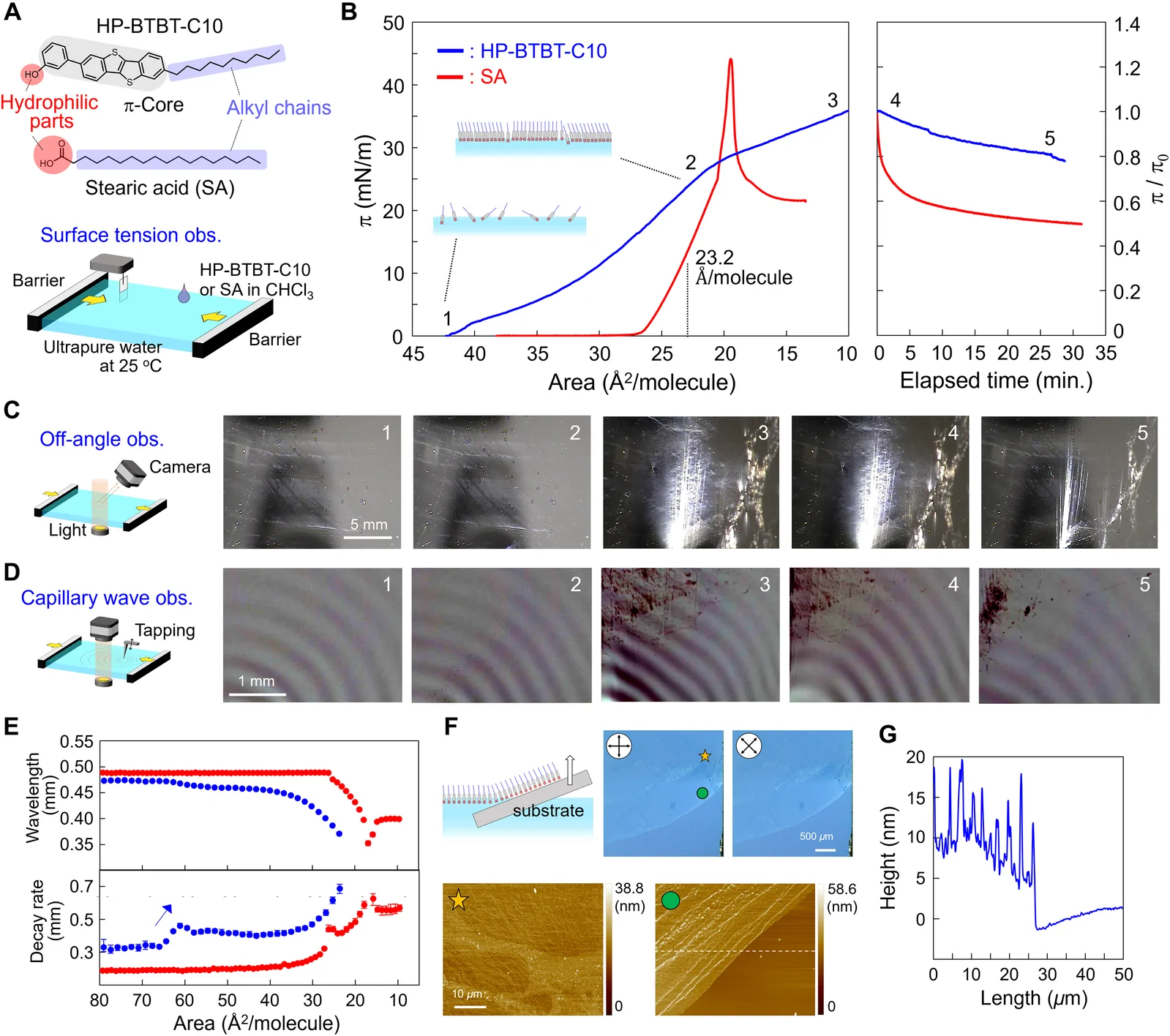
Self-folding and stretching behavior of an HP-BTBT-C10 monolayer at the air–water interface. (**A**) Molecular structures of HP-BTBT-C10 and stearic acid (SA), together with a schematic illustration of the Langmuir compression experiment. (**B**) Surface pressure–molecular area (π–A) isotherms (left) and temporal relaxation of surface pressure after full compression (right) for HP-BTBT-C10 (blue) and SA (red). HP-BTBT-C10 exhibits markedly slower relaxation than SA. (**C**) Off-axis optical micrographs and (**D**) capillary-wave images of HP-BTBT-C10 at the compression states indicated in (B). At high compression, linear white features aligned parallel to the barrier emerge in (C), accompanied by pronounced anisotropy and inhomogeneity in (D). (**E**) Molecular-area dependence of the capillary-wave wavelength (top) and decay length (bottom) for HP-BTBT-C10 (blue) and SA (red). The decay length of HP-BTBT-C10 increases slightly near ∼60 Å^2^ molecule^−1^, indicating that the monolayer remains condensed over a substantially broader density range than SA. (**F**) Schematic illustration of film transfer, crossed-Nicols optical micrograph of the transferred HP-BTBT-C10 film on glass, and AFM images of the regions indicated by the symbols in the optical micrograph. (**G**) Height profile extracted along the dashed line in the AFM image of (F).

HP-BTBT-C10 (Fig. 1A) is derived from the prototypical 2-decyl-7-phenyl[1]benzothieno[3,2-*b*][1]benzothiophene (Ph-BTBT-C10) framework (*17*, *18*) through hydroxy functionalization, which imparts amphiphilicity while preserving strong π–π interactions (*19*–*21*). In the bulk solid, HP-BTBT-C10 exhibits pronounced layered crystallinity and undergoes a transition to a layered liquid-crystalline phase at elevated temperatures (fig. S1). Recrystallization from organic solvents typically yields ultrathin plate-like single crystals, reflecting an intrinsic tendency to form ordered layered assemblies as determined by single-crystal structure analysis (fig. S2). As discussed below, these molecular characteristics provide the structural basis for mechanically adaptive behavior in the monolayer state.

We first examined the mechanical response of HP-BTBT-C10 monolayers at the air–water interface using Langmuir compression experiments. In contrast to stearic acid (SA), which exhibits a sharp rise in surface pressure followed by collapse (*5*–*10*), HP-BTBT-C10 and its longer chain derivatives (HP-BTBT-C14 and HP-BTBT-C18) display a gradual, continuous pressure increase over a wide molecular-area range (Fig. 1B and fig. S3). No abrupt failure occurs even at high surface pressures, indicating that the monolayer accommodates compression through internal rearrangement rather than simple densification and collapse. Time-dependent measurements further show that HP-BTBT-C10 retains a substantial fraction of applied stress over extended periods, whereas SA and Ph-BTBT-C10 relax rapidly (fig. S4), demonstrating persistent stress storage without collapse in the HP-BTBT-C10 monolayer.

To elucidate the origin of this anomalous response, we performed in situ optical observation under identical compression conditions. Off-axis imaging reveals localized changes in surface reflectivity upon entering the adaptive regime, appearing as white linear features parallel to the barrier (Fig. 1C). Their visibility suggests out-of-plane deformation sufficiently large to generate appreciable optical scattering, likely on the order of several tens to ∼100 nm. These features disappear after barrier motion stops and reappear upon recompression (fig. S5), demonstrating reversible formation and relaxation of large-scale interfacial structures.

### Capillary-wave imaging of out-of-plane deformation

Capillary-wave imaging (*26*–*28*) further shows that compression amplifies surface fluctuations (fig. S6). At low density, waves propagate isotropically in circular patterns, whereas pronounced anisotropy and inhomogeneity develop as the density approaches the critical packing area, localizing into discrete deformations (Fig. 1D). In contrast, SA monolayers preserve circular symmetry throughout. These observations indicate that HP-BTBT-C10 monolayers access additional out-of-plane degrees of freedom that are absent in conventional amphiphilic films.

Quantitative analysis of the capillary-wave wavelength λ_cw_ and decay length ξ_cw_ (Fig. 1E and fig. S7) shows that both systems exhibit a slight increase in ξ_cw_ upon monolayer compression, but HP-BTBT-C10 maintains this condensed state over a much broader density range than SA. These compressed states and the accompanying out-of-plane deformation coincide with the regime of continuous pressure increase and long-term stress retention in the π–*A* isotherms, suggesting a close link between stress accommodation and out-of-plane deformation. Transferred HP-BTBT-C*n* films on a substrate display inhomogeneous wrinkle-like optical patterns and weak birefringence under crossed Nicols (Fig. 1F and fig. S8). AFM confirms pronounced unidirectional bank-like morphologies corresponding to the wrinkle-like features (Fig. 1G).

### Electron diffraction and HRTEM analysis of folded multilayer wrinkles

**Fig. 2.**
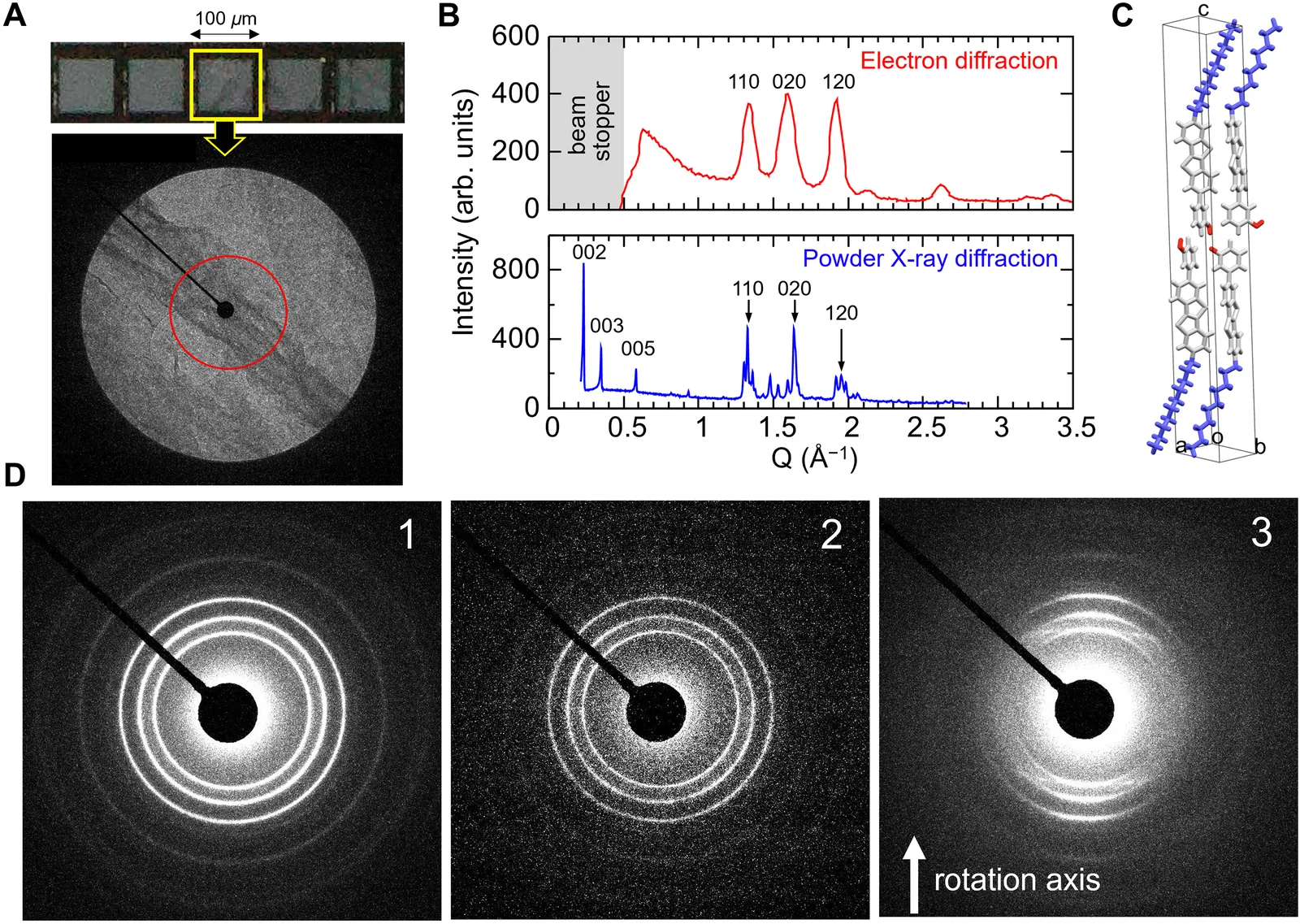
Electron diffraction analysis of self-folded HP-BTBT-C10 films. (**A**) A typical electron microscopy image of an HP-BTBT-C10 film transferred onto a copper grid coated with an ultrathin carbon film. A wrinkle traversing the film center is clearly visible. (**B**) Radially averaged electron diffraction intensity of the transferred film plotted as a function of scattering vector Q (upper), with powder x-ray diffraction of recrystallized HP-BTBT-C10 shown for comparison (lower). (**C**) Molecular packing structure of HP-BTBT-C10 determined by single-crystal x-ray diffraction. (**D**) Two-dimensional electron diffraction patterns acquired from the transferred film with the electron incident on (1) the entire area shown in (A), (2) the region enclosed by the red circle in (A), and (3) the same region while tilting the sample about an axis perpendicular to the beam, enhancing scattering along the tilt axis. These patterns demonstrate identical two-dimensional lattice ordering in both flat and wrinkled regions.

We next examined the structural consequences of out-of-plane deformation using electron diffraction (ED) (Fig. 2) (*29*). The ED patterns of the transferred films exhibit well-defined features corresponding to the (110), (020), and (120) reflections observed in powder x-ray diffraction (Fig. 2B). These results indicate that the intralayer herringbone packing motif (Fig. 2C and fig. S2) is at least locally retained in the transferred films. The ED patterns show isotropic Debye-Scherrer rings, implying that long-range orientational order is not preserved throughout the film. Spatially resolved and tilt-dependent diffraction data indicate that the characteristic local packing motif is retained over finite nanoscale domains in both flat and wrinkle-like regions (Fig. 2D and fig. S9). However, ED provides only spatially averaged structural information for the transferred films.

**Fig. 3.**
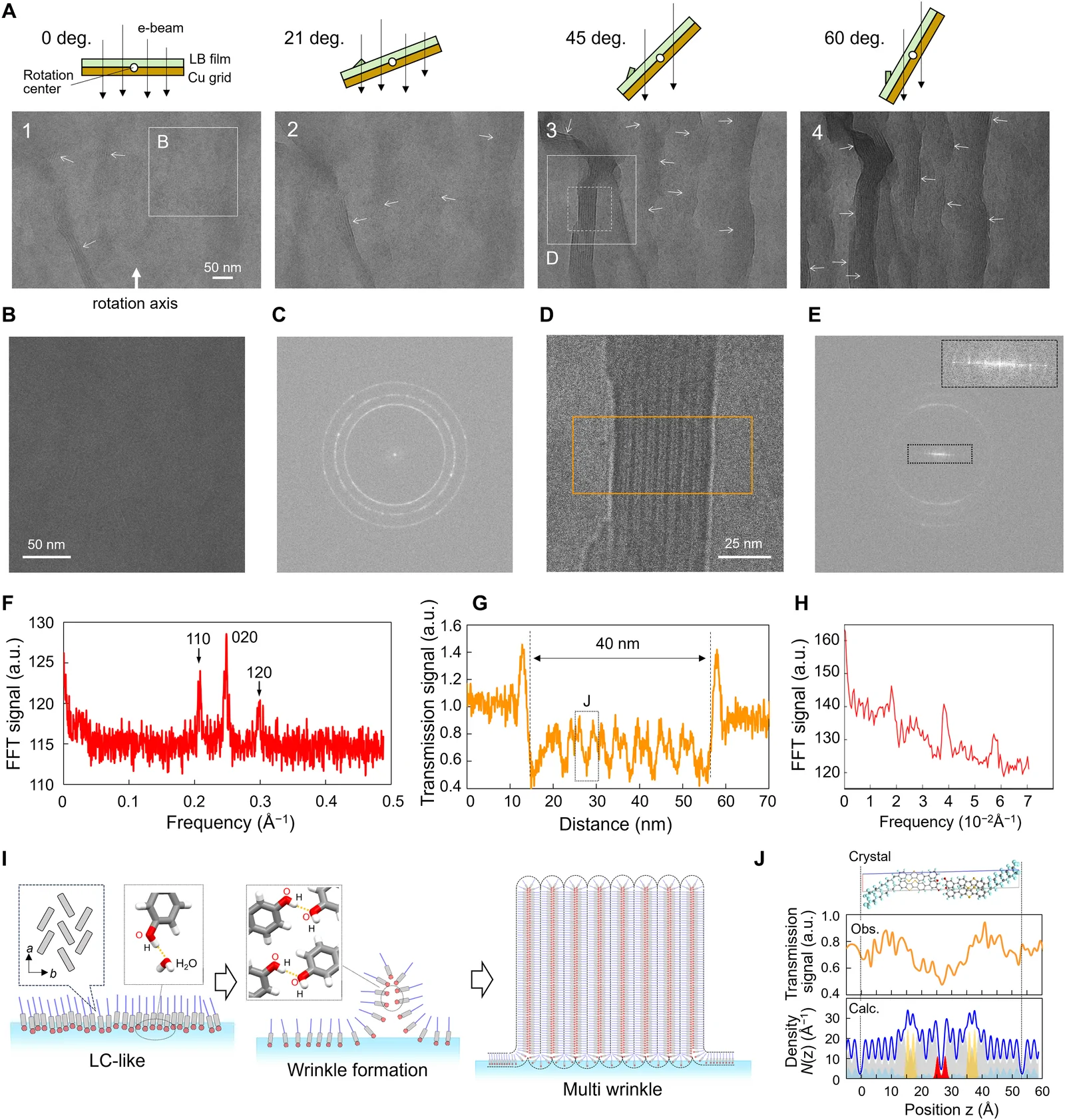
High-resolution transmission electron microscopy of self-folded HP-BTBT-C10 monolayer films. (**A**) HRTEM images of a transferred HP-BTBT-C10 film extracted from an aligned tilt series at sample tilt angles of 0°, 21°, 45°, and 60°. Wrinkles are indicated by white arrows. (B to H) Magnified views, FFT analyses, and intensity profiles of the HRTEM images. (**B**) Magnified view of the area enclosed by the white square in (A) at 0°. (**C**) FFT pattern of the image shown in (B). (**D**) Magnified view of the area enclosed by the dashed white square in (A) at 45°. (**E**) FFT pattern of the image shown in (D), with an expanded view of the central region shown in the inset (black rectangle). (**F**) Radially averaged FFT intensity in (C), showing clear peaks arising from the in-plane lattice periodicity. (**G**) TEM intensity profile taken perpendicular to the wrinkle axis in (D). (**H**) Directionally averaged FFT intensity taken perpendicular to the wrinkle axis in (D), showing a distinct peak corresponding to the wrinkle stacking periodicity. The HRTEM images in (A), (B), and (D) were gamma-corrected to enhance wrinkle contrast; see movie S1 for the corresponding images processed using brightness scaling only, without gamma correction. (**I**) Schematic illustration of a possible mesoscale wrinkle-formation process inferred from the experimental observations and the proposed stabilization of the HP-BTBT-C10 Langmuir monolayer on water and multilayer wrinkles through reorganization of hydrogen-bonding interactions. This schematic does not represent directly observed molecular trajectories or directly resolved local molecular arrangements, particularly at the edges of multilayers. (**J**) Comparison of the TEM intensity profile in (G) with the calculated electron-density profile determined from the crystal structure of HP-BTBT-C10.

We further examined the structural consequences of out-of-plane deformation by directly visualizing the monolayer and wrinkle-like regions using high-resolution transmission electron microscopy (HRTEM) equipped with a direct electron detector (*30*) and a cold field-emission electron source (*31*). Incidence-angle-dependent HRTEM revealed that the images consist mainly of nearly uniform regions, accompanied by several stripe-like domains that appear and disappear depending on the incidence angle (Fig. 3A and movie S1).

HRTEM and FFT analyses of the transferred films revealed both locally ordered flat regions and folded wrinkle-like regions (Fig. 3, B to H). Notably, fast Fourier transform (FFT) analysis of the uniform regions shows clear Debye–Scherrer rings corresponding to the (110), (020), and (120) diffraction features, as observed in the ED images (Fig. 3C). These results indicate that the uniform regions are composed of crystallites retaining the layered herringbone packing motif, with the molecular layer plane largely parallel to the film plane.

Among several stripe-like domains, a prominent one was selected for the magnified image and intensity-profile analysis shown in Fig. 3, D and G. Detailed analysis of the incidence-angle-dependent images reveals that this domain can be assigned to a wrinkle composed of stacked molecular layers, corresponding to 16 monolayers, or 8 bilayers, with a height of ∼40 nm (fig. S10 and movie S2). FFT analysis of this region reveals low-frequency features at ∼0.018 Å^−1^, corresponding to a wrinkle periodicity of ∼5.5 nm (Fig. 3H). Statistical analysis of numerous stripe-like regions gives a similar periodicity (5.4–5.8 nm), roughly corresponding to the thickness of a molecular bilayer (fig. S11). These observations support the interpretation that the HP-BTBT-C10 Langmuir monolayer on water undergoes a monolayer-to-wrinkle transformation through multiple-bilayer formation under lateral compression.

### Hydroxy group effects on monolayer and multilayer stabilization

To examine the role of the hydroxy groups, we performed intermolecular interaction calculations using dispersion-corrected density functional theory (DFT) (fig. S12) (*19*, *24*). The calculations show that HP-BTBT-C10 can form a hydrogen bond with a water molecule through the hydroxy group, which likely contributes to stabilizing the monolayer at the water surface, whereas such an interaction is absent for Ph-BTBT-C10. We also examined intermolecular interactions between HP-BTBT-C10 molecules based on the single-crystal structure. The calculations indicate that intermolecular hydroxy–hydroxy interactions across molecular layers can contribute to the stabilization of molecular bilayers. Thus, the hydroxy group may help stabilize both the monolayer at the water surface and the folded multilayer wrinkle structures.

The schematic diagram in Fig. 3I illustrates a possible mesoscale folding process during transformation between these two stabilized states under lateral compression, as inferred from experimental observations and supported by computational results. This schematic does not represent directly observed molecular trajectories or directly resolved local molecular arrangements, particularly at the edges of multilayers. Importantly, multilayer wrinkle formation, likely facilitated by the strong layered-ordering tendency of HP-BTBT-C10, provides a deformation pathway that can relieve compressive stress without catastrophic monolayer collapse, in striking contrast to conventional amphiphilic monolayers. The electron-density distribution determined from the crystal structure of HP-BTBT-C10 is also in good agreement with the transmission-signal profile obtained from the HRTEM images (Fig. 3J).

### Dynamic barrier-oscillation analysis of viscoelastic response

**Fig. 4.**
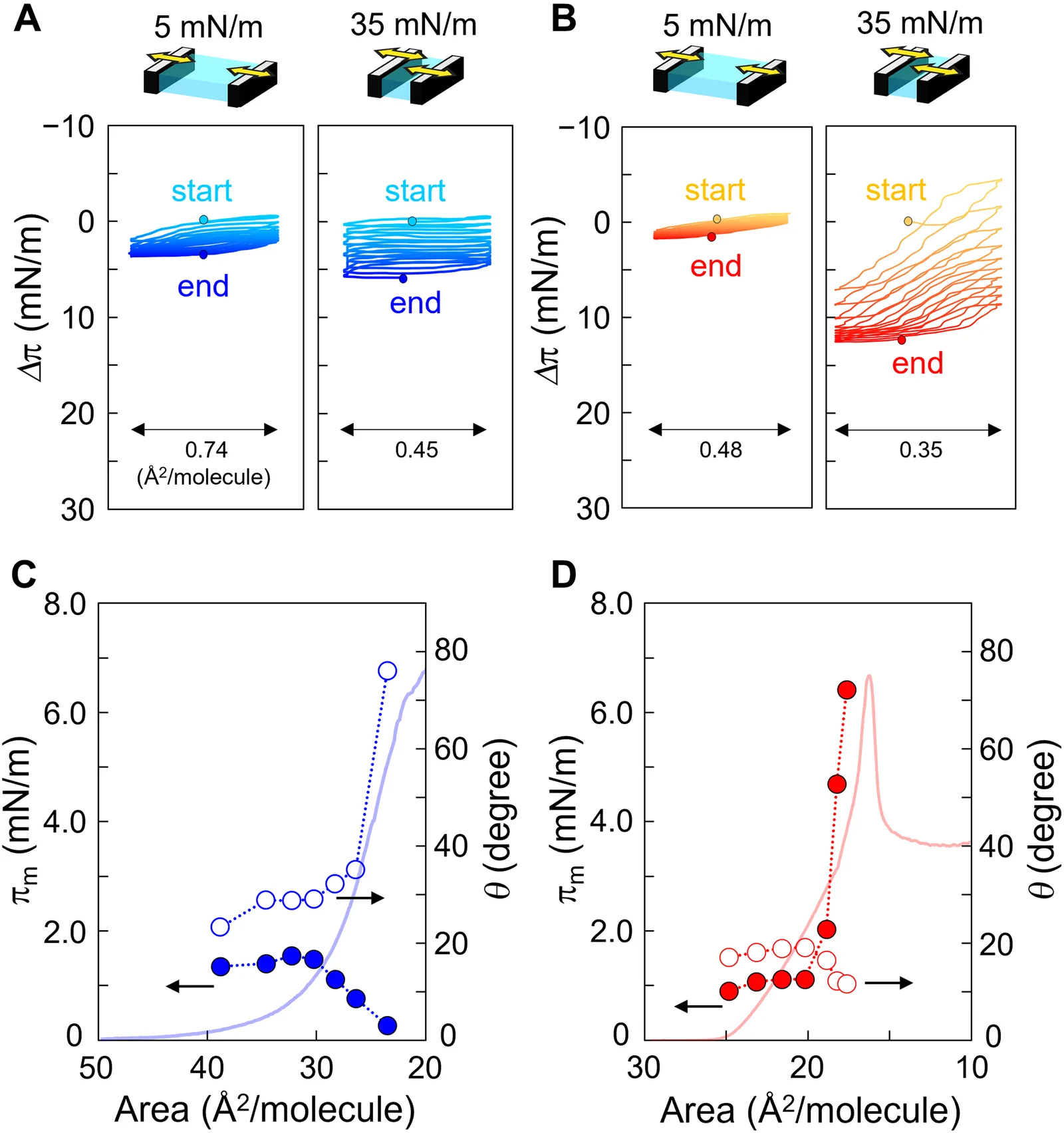
Dynamic viscoelastic response of adaptive and non-adaptive monolayers. (**A**) Representative surface-pressure responses at low (left) and high (right) mean surface pressures obtained from barrier-oscillation experiments for an HP-BTBT-C10 monolayer, and (**B**) corresponding responses for an SA monolayer. (**C**) Oscillation amplitude (π_m_, solid circles) and phase lag (θ, open circles) extracted from barrier-oscillation measurements for the HP-BTBT-C10 monolayer, plotted together with the corresponding π–A isotherm (solid curve), and (**D**) those for the SA monolayer. In contrast to SA, which undergoes monolayer collapse, HP-BTBT-C10 exhibits a pronounced decrease in pressure-modulation amplitude accompanied by a large increase in phase lag, reflecting viscoelastic stress accompanied via reversible folding and stretching.

To investigate the dynamic viscoelastic response of these monolayers at the water surface, we conducted dynamic barrier-oscillation experiments (Fig. 4 and fig. S13) (*32*–*34*). For the standard SA monolayer, the pressure-modulation amplitude (π_m_) increases considerably with increasing mean surface pressure, following the behavior of the π–*A* isotherm. However, this response changes significantly with repeated oscillations, accompanied by a large decrease in π_m_. In striking contrast, the HP-BTBT-C10 monolayer shows a decrease in π_m_ with increasing mean surface pressure, which does not follow the behavior of the π–*A* isotherm. Even under repeated oscillations, however, the modulation response itself remains stable, indicating a reproducible dynamic process. Analysis of the oscillation amplitude and phase lag extracted from these results reveals that the decrease in π_m_ for HP-BTBT-C10 is accompanied by a significant increase in phase lag, in sharp contrast to the response of the SA monolayer undergoing molecular collapse (Fig. 4, C and D and fig. S14).

These results reflect significant changes in viscoelastic stress associated with reversible folding and re-extension in HP-BTBT-C10. In particular, the density-dependent phase lag observed for HP-BTBT-C10 suggests a highly viscous regime characterized by reversible stress storage and release, rather than irreversible monolayer collapse. This behavior further suggests that the viscoelastic response is governed by folding and re-extension of the monolayer. Thus, wrinkle formation is not merely a static instability but a manifestation of a dynamic adaptive deformation process.

## DISCUSSION

These results reveal a distinctive mechanical response of molecular monolayers at water interfaces. Classical Langmuir monolayers typically behave as fragile two-dimensional molecular assemblies in which compression leads to solid-state densification and collapse, despite reported precursors of folding or wrinkling (*5*–*15*). In contrast, HP-BTBT-C10 monolayers undergo density-dependent structural transformations, folding into multilayers and re-extending into a monolayer. This behavior enables adaptation to mechanical stress through dynamic conversion of in-plane compression into out-of-plane deformation via compression-induced monolayer-to-multilayer structural reorganization.

The rod-like amphiphilic π-conjugated molecules presented in this study are characterized by a strong tendency to form layered molecular assemblies, arising from combined intermolecular interactions among the extended π-conjugated cores and linear alkyl chains (*18*–*25*). However, in the case of typical Ph-BTBT-C*n* molecules, the monolayer state at the water surface is unstable, and the molecules readily form bulk-like aggregates (see the inset in fig. S4B). This comparison indicates that the hydroxy group plays a crucial role in stabilizing the HP-BTBT-C10 Langmuir monolayer at the air–water interface.

Importantly, HP-BTBT-C10 exhibits a crystal structure closely resembling that of Ph-BTBT-C10. The unit-cell parameters of these compounds are similar, and the intermolecular arrangements of the π-conjugated cores and alkyl substituents are also largely preserved (fig. S2). These results suggest that meta-substitution of the phenyl group with a hydroxy group introduces hydrogen-bonding interactions without substantially disrupting the intrinsic layered packing motif of Ph-BTBT-C10. This structural compatibility likely allows HP-BTBT-C10 to retain the strong layered-ordering tendency characteristic of Ph-BTBT-C10 while acquiring amphiphilicity and hydrogen-bonding capability. Nevertheless, systematic comparison with positional isomers will be necessary to establish more general molecular design rules for autonomous folding and mechanical adaptation.

We also point out that another plausible origin of this reversible out-of-plane deformation under compression is related to the liquid-crystalline nature of the molecular assembly (*17*, *20*, *22*, *23*), which may facilitate collective structural reorganization. The material exhibits a bulk liquid-crystalline phase at elevated temperature (fig. S1). Additionally, related rod-like molecules composed of extended π-conjugated cores and linear alkyl chains have been reported not only to exhibit bulk liquid-crystalline phases at high temperatures, but also to form lyotropic liquid-crystal-like layers at air-liquid interfaces from organic-solvent solutions (*20*–*25*). The enhanced molecular mobility associated with such liquid-crystal-like states may facilitate large-scale structural reorganization between the monolayers on water and the out-of-plane folded multilayer wrinkle structures. However, the liquid-crystalline nature of the present Langmuir monolayer has not been directly verified and should be examined in future studies.

Further systematic studies using more closely related control molecules, such as π-conjugated amphiphiles with different hydrophilic groups, substitution positions, or alkyl-chain architectures, will be important to establish the detailed molecular requirements for mechanical adaptability. More broadly, these findings bridge the conceptual gap between conventional molecular films and mechanically adaptive biological membranes. By demonstrating that a rationally designed molecular monolayer can accommodate compression through reversible folding, wrinkling, and re-extension while retaining local molecular packing, this work provides a design concept for mechanically adaptive two-dimensional soft materials at interfaces.

## MATERIALS AND METHODS

### Synthesis of HP-BTBT-C*n*

**Fig. 5.**
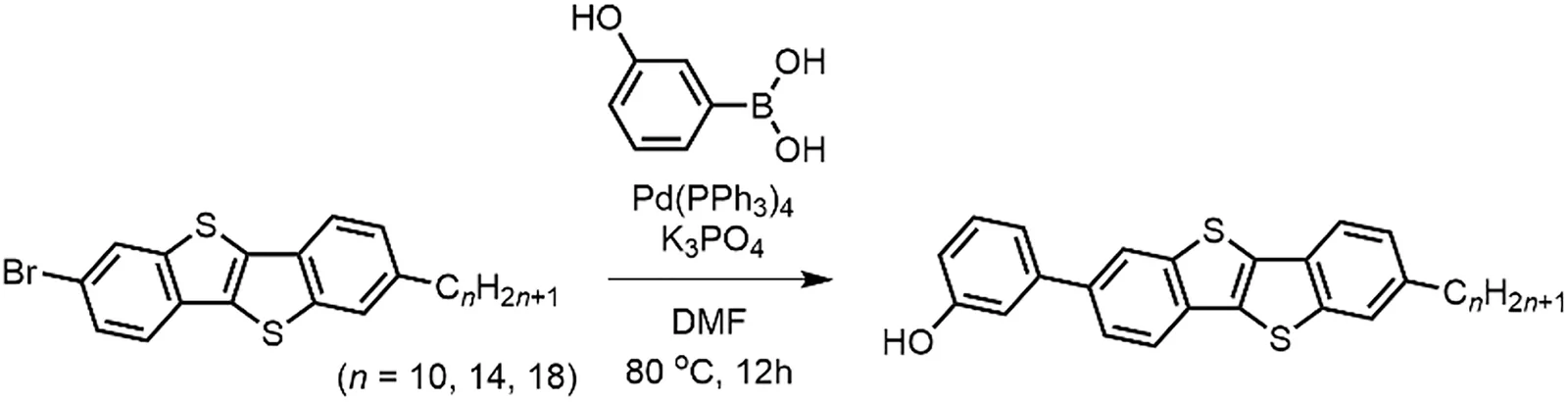
Synthetic scheme of HP-BTBT-C*n*.

HP-BTBT-C*n* (*n* = 10, 14, 18) was synthesized via a palladium-catalyzed Suzuki coupling reaction between the corresponding Br-BTBT-C*_n_* and 3-hydroxyphenylboronic acid, as shown in Fig. 5. The Br-BTBT-C*n* precursors were prepared according to a previously reported procedure (*18*).

Br-BTBT-C*n* (0.61 mmol), 3-hydroxyphenylboronic acid (421 mg, 3.05 mmol, 5.0 equiv), Pd(PPh_3_)_4_ (38 mg, 5 mol%), and K_3_PO_4_ (416 mg, 3.0 equiv) were dissolved in DMF (20 mL), and the mixture was stirred at 80°C for 12 h under nitrogen. After cooling to room temperature, the reaction mixture was poured into methanol to precipitate a gray solid, which was collected by filtration and washed with water. The crude product was dissolved in hot chloroform and passed through a silica gel pad (chloroform eluent). Removal of the solvent under reduced pressure afforded a white solid, which was further purified by recrystallization from chloroform/ethanol to yield HP-BTBT-C*n*.

### Crystal structure analysis of HP-BTBT-C10

A single crystal of HP-BTBT-C10 suitable for structural analysis was obtained by recrystallization from a saturated solution in anisole at room temperature. The crystal obtained from the solution was carefully transferred directly to a mounting device for structural analysis (LithoLoops, Protein Wave Corp.). Single-crystal x-ray diffraction (XRD) measurements were performed using a Rigaku AFC10 four-circle diffractometer equipped with a PILATUS 200 K hybrid pixel detector. Data correction and reduction were performed using CrysAlisPro (Rigaku Corp.). Refinement was carried out using the CrystalStructure software package (Rigaku Corp.). The initial structure was solved by direct methods using SIR2008 and refined by the full-matrix least-squares method using SHELXL, with anisotropic displacement parameters applied to non-hydrogen atoms. Hydrogen atoms were placed at geometrically calculated positions.

The crystallographic data for HP-BTBT-C10 have been deposited with the Cambridge Crystallographic Data Centre under CCDC deposition number 2553913.

### Langmuir film formations and observations

Surface pressure-area (π − A) isotherms and relaxation measurements were performed using a KSV NIMA Langmuir−Blodgett trough (Biolin Scientific). The subphase temperature was maintained at 25°C using an EYELA NCB-1220A external chiller. Ultrapure water obtained from a Millipore Milli-Q system was used as the subphase. The subphase pH was adjusted with NaHCO_3_ (TCI). Surface pressure was monitored using a Wilhelmy plate made of Kiriyama No. 5B filter paper.

For the formation of stearic acid Langmuir monolayer, a 0.5 mg mL^−1^ solution of stearic acid (TCI) in chloroform (TCI) was prepared and spread onto the subphase using a Hamilton microsyringe. After a 15 min incubation period to allow complete solvent evaporation, the barriers were compressed at a constant rate. Upon reaching the target surface pressure, barrier motion was halted, and surface pressure relaxation was recorded.

For the formation of HP-BTBT-C*n* (*n* = 10,14,18) Langmuir films, HP-BTBT-C*n* solutions (0.5 mg mL^−1^ in chloroform) were prepared by heating at 80°C for 10 min to ensure complete dissolution. The solutions were deposited onto the subphase using a microsyringe immediately after heating. Following a 7 min equilibration period, the barriers were compressed, and surface pressure relaxation was subsequently measured after stopping the barriers.

For in situ off-angle observation of the air−water interface during Langmuir film formation, a camera assembly consisting of a Sentech STC-620PWJ camera (Sony ICX418AKL, 1/2-inch interlaced CCD sensor) equipped with a Tokina KCM-Z084 lens was employed.

### Surface capillary wave experiments

To visualize surface capillary waves at the air−water interface, a high-speed imaging system was integrated with the Langmuir−Blodgett trough. The system consisted of a Keyence VW-2000 high-speed camera equipped with a VW-600Z camera unit and a VH-Z250WSPA macro lens. The xenon light source originally installed in the high-speed camera was used for illumination. To optimize the optical path for surface wave visualization, a light-shaping lens (Thorlabs LA4052, *f* = 35.1 mm) was mounted using an LMR1/M lens mount (Thorlabs). Optical alignment was carefully adjusted to enhance the contrast of shadows arising from surface tension waves.

Surface waves were generated using a handmade oscillator comprising a piezoelectric element (Thorlabs PK44LA2P2) coupled to a stainless-steel wire (SUS316, diameter of 0.5 mm; Nilaco). The piezoelectric element was driven by a sinusoidal signal generated by a function generator (AS ONE AWG1005) and amplified using a high-voltage amplifier (Toyo Technica A400). To prevent charge accumulation in the piezoelectric element, a 22 kΩ resistor (Takman RLF3SJ, 3 W) was connected in parallel.

Surface wave measurements were conducted at an oscillation frequency at 2.015 kHz, with a drive voltage of 100 V_pp_ applied to the mechanical transducer (5 V_pp_ input amplified by a factor of 20). High-speed imaging was performed at a frame rate of 30 fps.

### Electron diffractometry and high-resolution electron microscopy

Monolayer films were scooped from the water surface onto holey carbon films (Quantifoil R1/4; Quantifoil Micro Tools GmbH) supported on 200-mesh copper grids (Maxtaform HF34) and air-dried. The grids were transferred to a JEOL CRYO ARM 300 transmission electron microscope operated at an accelerating voltage of 300 kV. Rotational diffraction patterns were collected at room temperature on a DE64 direct electron detector (*35*) (DirectElectron), controlled by SerialEM (*36*) and ParallEM (*29*, *37*). The camera length was nominally set to 500 mm and calibrated from gold ring patterns.

For tomographic analysis, the samples were imaged at room temperature using a JEOL CRYO ARM 300II electron microscope operated at an accelerating voltage of 300 kV. Tilt series were collected using SerialEM (*36*) with an in-house-modified version of a FastTomo script (*38*, *39*) in a bidirectional scheme, from 0° to 60° and from −3° to −60°, with a step of 3°. The nominal magnification was set to 15,000× or 50,000× at defocus settings of −1, −2 or − 3 μm, and inelastically scattered electrons were removed through an in-column energy filter with a slit width of 20 eV. At each tilt angle, dose-fractionated images were recorded on a K3 direct electron detector (Gatan, AMETEK) in CDS mode at a dose rate of ∼2 e^−^ Å^−2^ or ∼3 e^−^ Å^−2^ for 15,000× or 50,000×, respectively. Image stacks were drift-corrected, dose-weighted, and summed using the MotionCor2 algorithm (*40*) implemented in RELION-3.1 (*41*). Motion-corrected tilt series were aligned using AreTomo (*42*).

The combination of a direct electron detector and a cold field emission electron gun enables high-resolution imaging of samples, such as lattice patterns of small organic molecular crystals (*39*), and the visualization of hydrogen atoms in protein molecules (*43*).

### Intermolecular interaction calculations

Atomic positions in the HP-BTBT-C10 and Ph-BTBT-C10 crystal were optimized using Quantum ESPRESSO (*44*) with the PBE functional (*45*) and the D3BJ dispersion correction (*46*), starting from the structural data obtained from single-crystal x-ray diffraction analysis. Gaussian16 (*47*) was employed for the geometry optimizations of the HP-BTBT-C10–water and Ph-BTBT-C10–water complexes, as well as for intermolecular interaction energy calculations at the PBE/6-311G** level with the D3BJ dispersion correction. The basis set superposition error (BSSE) was corrected using the counterpoise method for the intermolecular interaction energy calculations (*48*).

The intermolecular interaction energies were calculated using the optimized crystal geometries. For Ph-BTBT-C10, which has a single crystallographically independent molecule in the unit cell, the interaction energies between this molecule and neighboring molecules in the adjacent layer were evaluated. For HP-BTBT-C10, the interaction energies were calculated for each of the four crystallographically independent molecules with their respective neighboring molecules in the adjacent layer. In these calculations, only molecular pairs with a minimum interatomic distance of 8 Å or less were considered. In addition, the interaction energies of the HP-BTBT-C10–water and Ph-BTBT-C10–water complexes were calculated using the optimized geometries of the respective complexes.

### Barrier oscillation experiments

Barrier oscillation experiments were conducted at a frequency of 50 mHz with a relative area modulation amplitude of 1%. The resulting surface pressure data (raw data shown in fig. S11) were analyzed using ten oscillation cycles for each barrier oscillation experiment. Upon completion of each measurement, the system was immediately advanced to the next target surface pressure.

The data were fitted using the following phenomenological equation;

$$\pi =\pi_{0}e^{-\mathrm{\alpha t}}+\pi_{m}e^{-\mathrm{\beta t}}\text{sin}\left(\mathrm{\omega t}-\theta \right)$$ (1)

where π(*t*) is the surface pressure, *t* is time, and ω is the angular frequency of the barrier oscillation. Here, π_m_ represents the initial mean surface pressure, π_m_ is the oscillation amplitude, α and β are the decay constants for the baseline and oscillatory components, respectively, and θ denotes the phase retardation.

## Supplementary Materials

### The PDF file includes:

Note S1 Figs. S1 to S14 Legends for movies S1 and S2
