# Supplementary material for: Self-stretching molecular monolayers with autonomous folding and mechanical adaptation
Source: Sci Adv. 2026 Sep 9;12(37):eaeg8906. doi: 10.1126/sciadv.aeg8906 (PMC13557093; doi:10.1126/sciadv.aeg8906)
Supplement: Supplementary file 1 [file sciadv.aeg8906_sm.pdf]

Science Advances
AAAS

Supplementary Materials for

Self-stretching molecular monolayers with autonomous folding and mechanical adaptation

Taichi Iizuka *et al.*

Corresponding author: Taichi Iizuka, t-iizuka@classnk.or.jp; Tatsuo Hasegawa, t-hasegawa@ap.t.u-tokyo.ac.jp

Sci. Adv. **12**, eaeg8906 (2026)
DOI: 10.1126/sciadv.aeg8906

The PDF file includes:

Note S1
Figs. S1 to S14
Legends for movies S1 and S2

Other Supplementary Material for this manuscript includes the following:

Movies S1 and S2

Supplementary Note S1

Characterization data of newly synthesized compounds

HP-BTBT-C10: colorless crystal (32% yield). ^{1}H-NMR (400 MHz, $CDCl_3$): δ (ppm) 8.10 (s, 1H, ArH), 7.91 (d, *J* = 8.4 Hz, 1H, ArH), 7.79 (d, *J* = 8.4 Hz, 1H, ArH), 7.73 (s, 1H, ArH), 7.67 (dd, *J* = 8.4, 1.0 Hz, 1H, ArH), 7.35 (dd, *J* = 8.4, 8.4 Hz, 1H, ArH), 7.29 (m, 2H, ArH), 7.16 (m, 1H, ArH), 6.85 (dd, *J* = 8.4, 1.0 Hz, 1H, ArH), 4.86 (br, 1H, OH), 2.77 (t, *J* = 8.0 Hz, 2H, CH_2), 1.70 (tt, *J* = 8.0, 7.2 Hz, 2H, CH_2), 1.35-1.27 (br, 14H, CH_2), 0.88 (t, *J* = 7.2 Hz, 3H, CH_3). ^{13}C-NMR (150MHz, $CDCl_3$, 55°C): δ (ppm) 156.08, 142.98, 142.84, 142.71, 140.57, 137.72, 133.92, 132.68, 132.47, 131.19, 130.15, 126.02, 124.42, 123.41, 122.36, 121.56, 121.29, 120.04, 114.44, 114.35, 36.18, 31.91, 31.62, 29.63, 29.60, 29.53, 29.32, 22.67, 14.03. HRMS (APCI) m/z calcd for $C_{30}H_{32}OS_2^+$ $(M+H)^+$ 473.1967, found 473.2019.

HP-BTBT-C14: colorless crystal (37% yield). ^{1}H-NMR (400 MHz, $CDCl_3$): δ (ppm) 8.10 (s, 1H, ArH), 7.91 (d, *J* = 8.4 Hz, 1H, ArH), 7.79 (d, *J* = 8.4 Hz, 1H, ArH), 7.73 (s, 1H, ArH), 7.67 (dd, *J* = 8.4, 1.0 Hz, 1H, ArH), 7.35 (dd, *J* = 8.4, 8.4 Hz, 1H, ArH), 7.29 (m, 2H, ArH), 7.16 (m, 1H, ArH), 6.85 (dd, *J* = 8.4, 1.0 Hz, 1H, ArH), 4.86 (br, 1H, OH), 2.77 (t, *J* = 8.0 Hz, 2H, CH_2), 1.70 (tt, *J* = 8.0, 7.2 Hz, 2H, CH_2), 1.35-1.27 (br, 22H, CH_2), 0.88 (t, *J* = 7.2 Hz, 3H, CH_3). ^{13}C-NMR (150MHz, $CDCl_3$, 55°C): δ (ppm) 156.09, 143.00, 142.86, 142.73, 140.57, 137.73, 133.93, 132.69, 132.48, 131.20, 130.13, 126.01, 124.42, 123.39, 122.36, 121.54, 121.29, 120.04, 114.44, 114.36, 36.17, 31.92, 31.59, 29.65, 29.58, 29.50, 29.33, 29.31, 22.66, 14.00. HRMS (APCI) m/z calcd for $C_{34}H_{40}OS_2^+$ $(M+H)^+$529.2593, found 529.2618.

HP-BTBT-C18: colorless crystal (37% yield). ^{1}H-NMR (400 MHz, $CDCl_3$): δ (ppm) 8.10 (s, 1H, ArH), 7.91 (d, *J* = 8.4 Hz, 1H, ArH), 7.79 (d, *J* = 8.4 Hz, 1H, ArH), 7.73 (s, 1H, ArH), 7.67 (dd, *J* = 8.4, 1.0 Hz, 1H, ArH), 7.35 (dd, *J* = 8.4, 8.4 Hz, 1H, ArH), 7.29 (m, 2H, ArH), 7.16 (m, 1H, ArH), 6.85 (dd, *J* = 8.4, 1.0 Hz, 1H, ArH), 4.86 (br, 1H, OH), 2.77 (t, *J* = 8.0 Hz, 2H, CH_2), 1.70 (tt, *J* = 8.0, 7.2 Hz, 2H, CH_2), 1.35-1.27 (br, 30H, CH_2), 0.88 (t, *J* = 7.2 Hz, 3H, CH_3). ^{13}C-NMR (150MHz, $CDCl_3$, 55°C): δ (ppm) 156.09, 143.00, 142.86, 142.73, 140.54, 133.93, 132.70, 132.38, 131.20, 130.13, 126.00, 124.42, 123.36, 122.36, 121.54, 121.29, 120.04, 114.44, 114.36, 36.17, 31.92, 31.59, 29.68, 29.65, 29.58, 29.50, 29.33, 29.31, 22.66, 14.00. HRMS (APCI) m/z calcd for $C_{38}H_{48}OS_2^+$ $(M+H)^+$ 585.3219, found 585.3227.

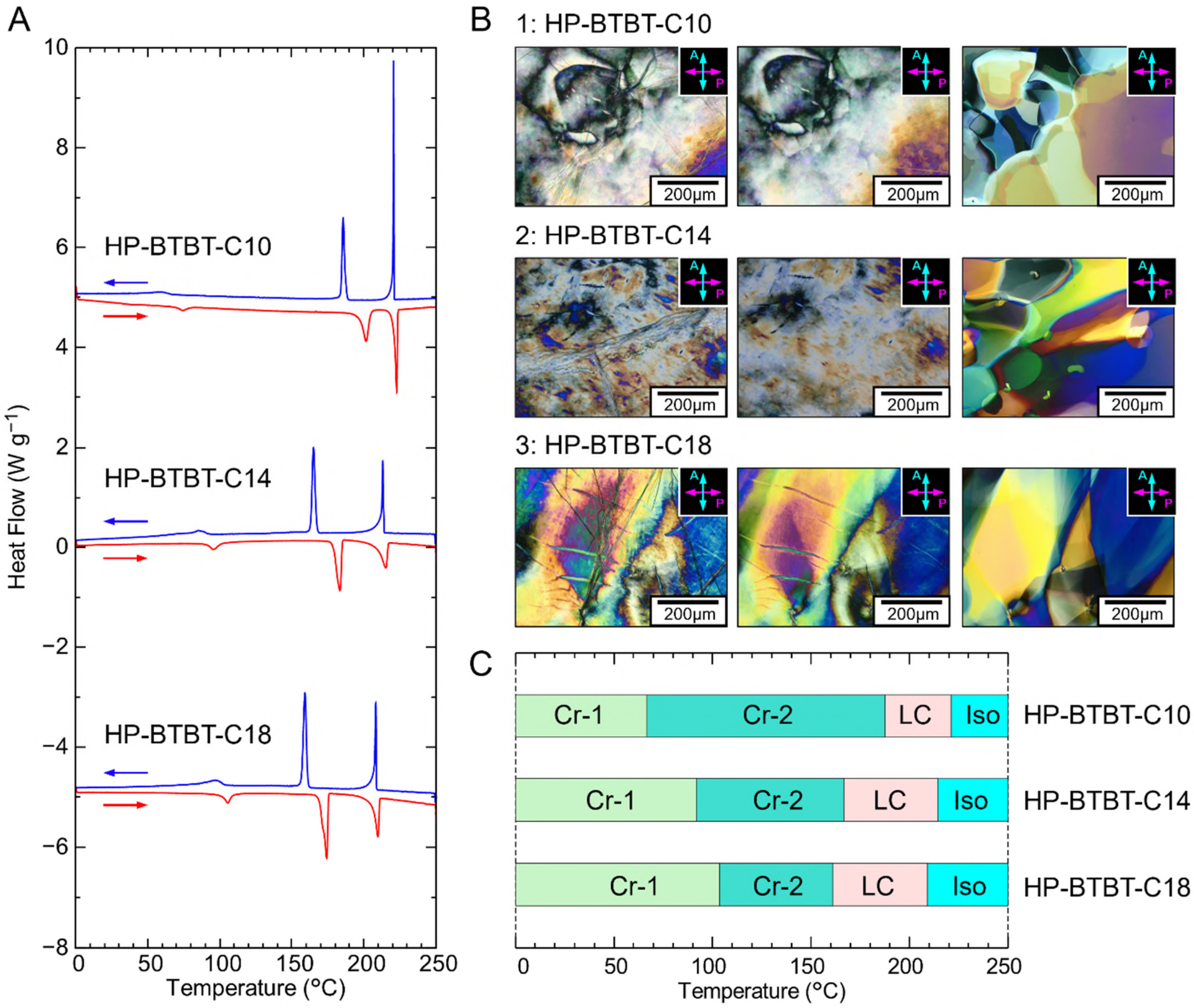

Fig. S1. Temperature-induced phase transitions in HP-BTBT-C*n*. (A) Differential scanning calorimetry (DSC) thermograms of HP-BTBT-C*n* (*n* = 10, 14, 18) recorded during the second heating and cooling cycle at a scan rate of 5 °C min^{-1}. The curves are vertically offset for clarity. **(B)** Polarized optical microscopy (POM) images of HP-BTBT-C*n* (*n* = 10, 14, 18) acquired during cooling, showing a mosaic texture at 200 °C. **(C)** Phase diagrams of HP-BTBT-C*n* (*n* = 10, 14, 18) constructed from DSC and POM observations. Cr, LC and Iso denote the solid crystalline, liquid crystalline, and isotropic liquid phases, respectively. Phase transition temperatures were determined from the cooling curves in the DSC measurements.

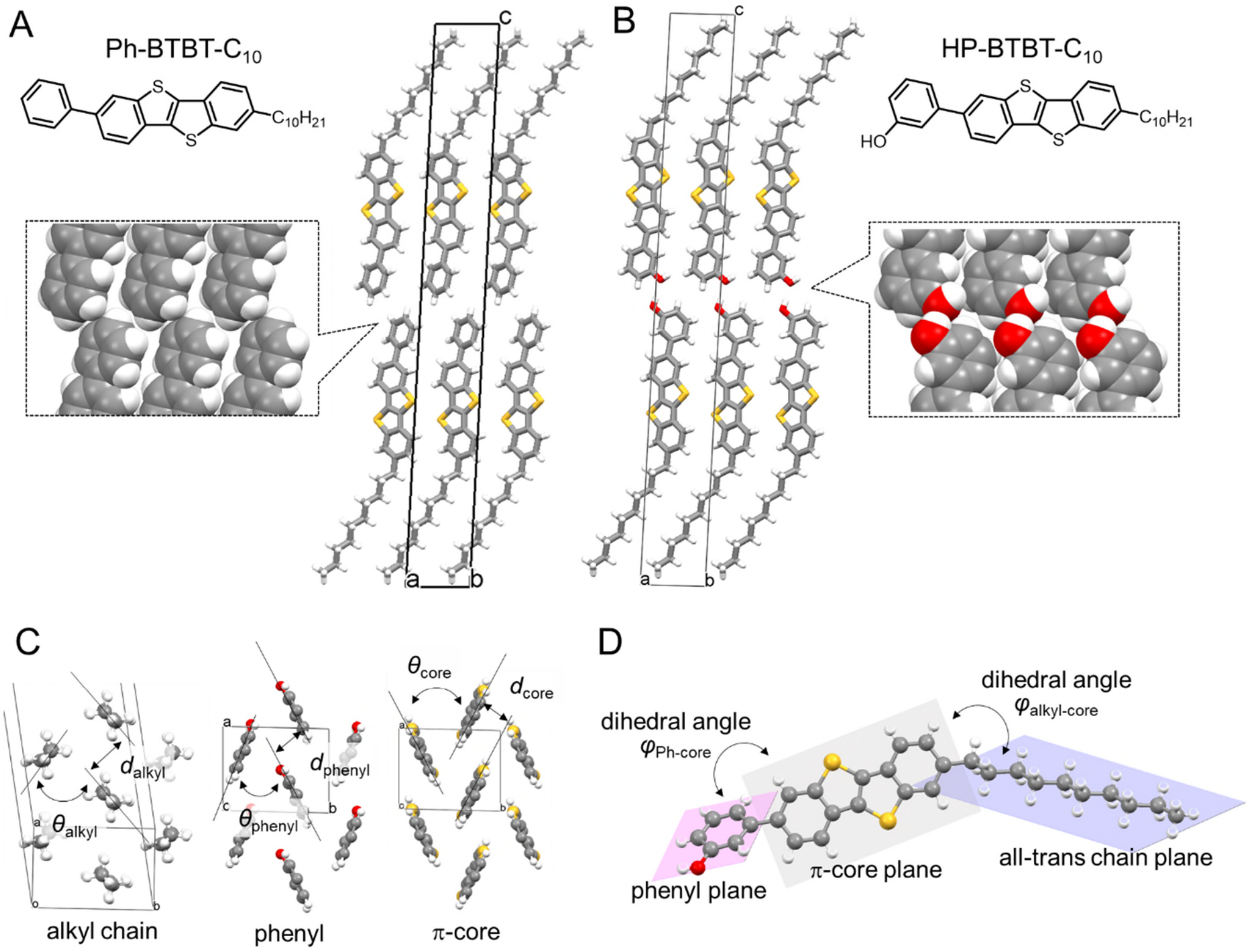

E

Lattice constant

	Space group	a (Å)	b (Å)	c (Å)	α (deg.)	β (deg.)	γ (deg.)
HP-BTBT-C10	$P1$	6.0788 (3)	7.6471 (2)	53.725 (2)	92.923 (2)	91.517 (3)	91.234 (3)
Ph-BTBT-C10[S1]	$P2_1/a$	6.0471 (3)	7.7568 (4)	53.124 (5)	–	93.135 (3)	–

Intralayer arrangement

	herringbone angle θ (deg.)			herringbone distance d (Å)			dihedral angle φ (deg.)	
	θ_{alkyl}	θ_{phenly}	θ_{core}	d_{alkyl}	d_{phenyl}	d_{core}	$\varphi_{Ph\text{-}core}$	$\varphi_{alkyl\text{-}core}$
HP-BTBT-C10	85.0	46.1	49.2	3.75	2.48	2.52	48.8	66.4
Ph-BTBT-C10	81.1	86.5	50.3	3.35	4.05	2.57	19.6	62.9

Fig. S2. Crystal packing of HP-BTBT-C10 in comparison with Ph-BTBT-C10. Crystal packing structures of **(A)** Ph-BTBT-C10 and **(B)** HP-BTBT-C10, projected along the *a*-*c* plane. Space-filling models are included to highlight the interlayer arrangements. **(C)** Intralayer arrangements of alkyl chain layer, phenyl layer, and π-core layer, along with the definition of herringbone angle θ and distance d. **(D)** Definition of dihedral angle φ. **(E)** Summary of lattice constants and intralayer arrangement parameters for Ph-BTBT-C10 and HP-BTBT-C10.

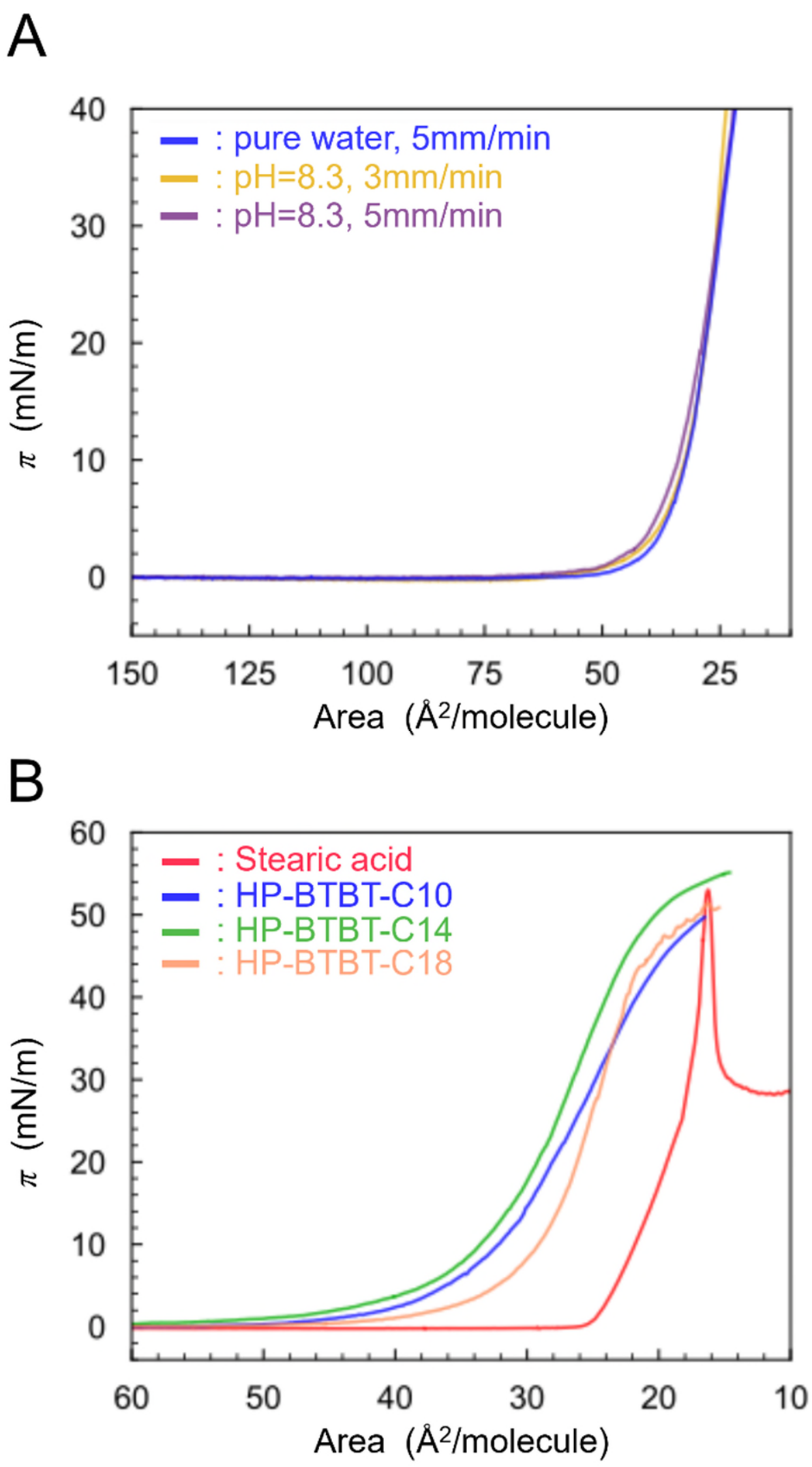

Fig. S3. π−A isotherms of HP-BTBT-C*n* measured under different conditions. (**A**) π−A isotherms of HP-BTBT-C10 measured at different barrier compression speeds (3 and 5 mm min^{-1}) and under different subphase conditions (pure water and pH 8.3). The subphase pH was adjusted to 8.3 by adding $NaHCO_3$ to pure water. Under the pH 8.3 condition, isotherms obtained at barrier speeds of 3 mm min^{-1} (brown curve) and 5 mm min^{-1} (purple curve) are shown. For comparison, the π−A isotherm measured on pure water at a barrier speed of 5 mm min^{-1} is also shown (blue curve). (**B**) π−A isotherms of stearic acid (red curve) and HP-BTBT-C*n* with different alkyl chain lengths (*n* = 10, blue curve; *n* =14, green curve; *n* =18, orange curve), measured on pure water at a barrier speed of 5 mm min^{-1}.

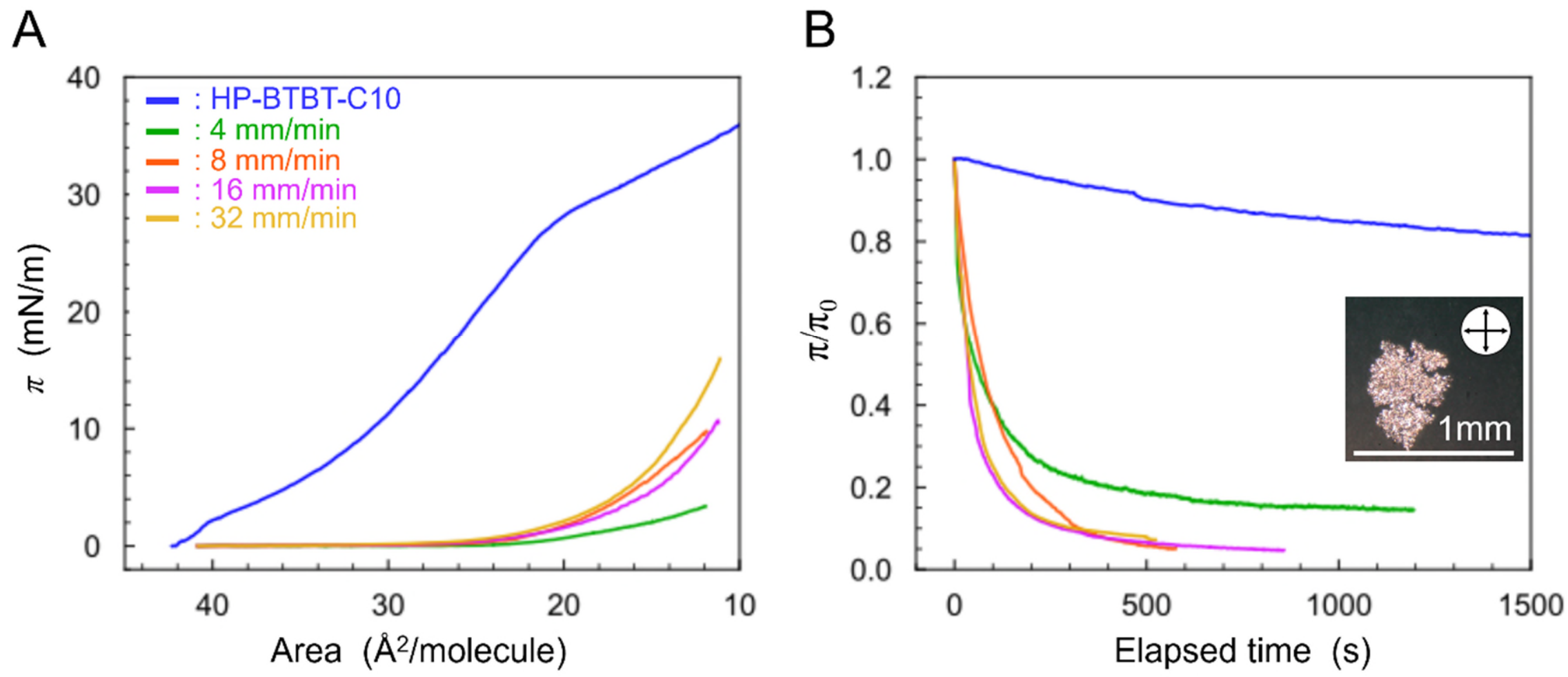

Fig. S4. Application of the Langmuir technique to Ph-BTBT-C10. (A) π−A isotherms of Ph-BTBT-C10 on pure water, measured at different barrier compression speeds (4 mm min^{-1}, green curve; 8 mm min^{-1}, orange curve; 16 mm min^{-1}, purple curve; 32 mm min^{-1}, brown curve). For comparison, the π−A isotherm of HP-BTBT-C10 measured on pure water at a barrier speed of 8 mm min^{-1} is also shown (blue curve). These results clearly demonstrate that the introduction of a hydroxyl group significantly alters the stability of the Langmuir monolayer. **(B)** Time-dependent evolution of surface pressure after the barrier was compressed to approximately 11 $Å^2$ $molecule^{-1}$, as indicated in (A). The surface pressure is normalized to the value at the end of compression. The inset shows a photograph of the aggregation of fine crystals observed following a substantial decrease in surface pressure.

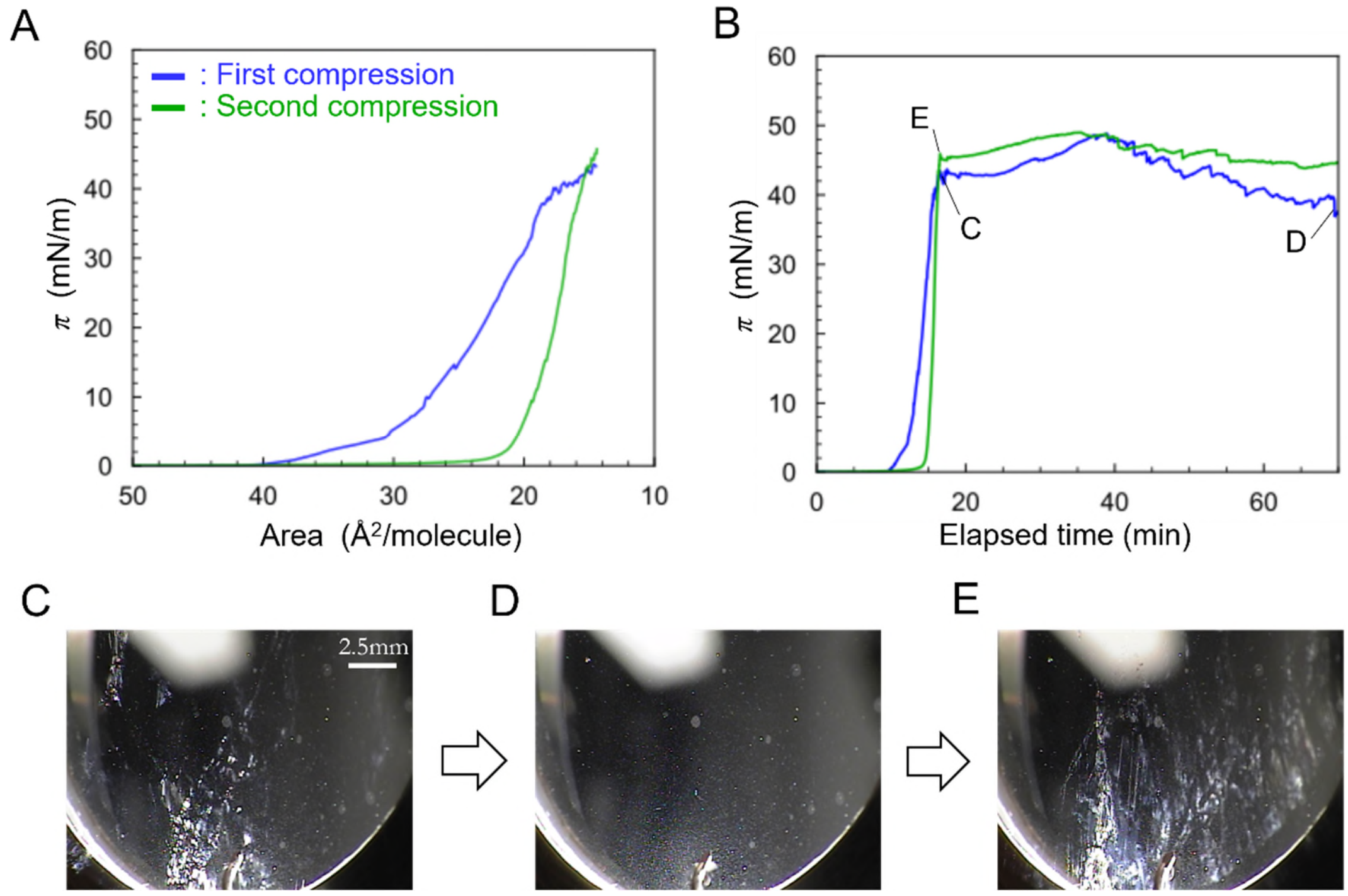

Fig. S5. Cycle- and time-dependent variation of surface pressure and morphology. (A) π−A isotherms of the HP-BTBT-C10 Langmuir monolayer recorded during the first (blue curve) and second (green curve) compression cycles at a barrier speed of 8 mm min^{-1} (subphase pH = 8.3, adjusted with $NaHCO_3$). The plot also includes the time-dependent evolution of surface pressure over 1 h after stopping barrier motion near 15 $Å^2$ $molelcule^{-1}$. **(B)** Time evolution of surface pressure replotted from (A). (**C−E**) Photographs of the monolayer taken **(C)** immediately after the first compression, **(D)** after sufficient relaxation, and **(E)** immediately after the second compression. These results indicate that the folded molecular layers gradually relax and re-extend over time, accompanied by an increase in surface pressure, demonstrating a self-organized two-dimensional rearrangement of the molecules.

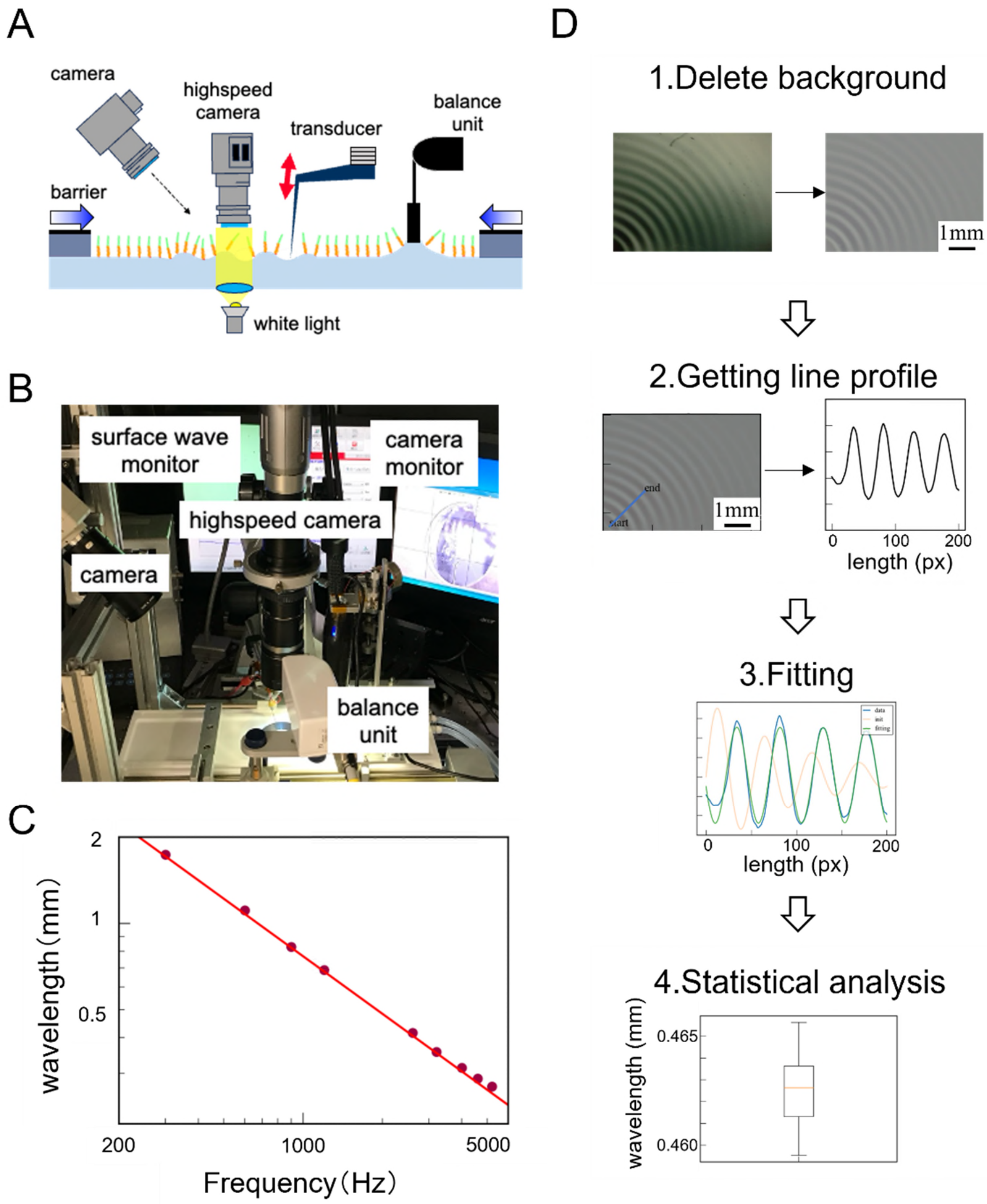

Fig. S6. Capillary wave observation technique. (A) Schematic illustration and **(B)** photograph of the experimental setup used in this study, **(C)** Plot of wavelength versus frequency for surface waves on pure water, measured using the setup shown in (A) and (B). The surface tension of water was taken to be 72 mN m^{-1}, and the red solid line represents the theoretical relationship based on the Kelvin equation. **(D)** Schematic illustration of the capillary wave analysis procedure used to extract the wavelength and decay length.

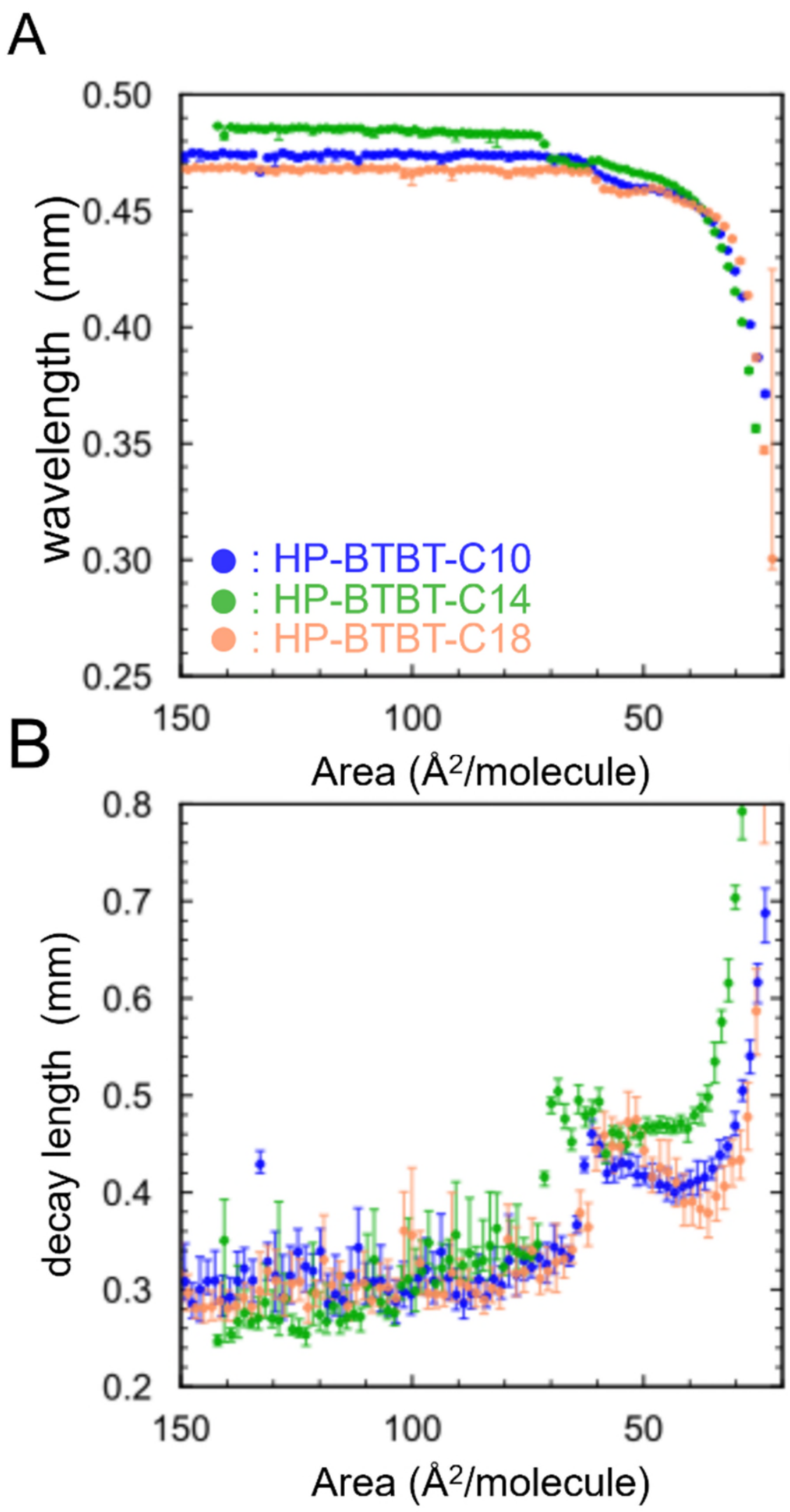

Fig. S7. Results of capillary wave analysis for water surfaces covered with HP-BTBT-C*n* Langmuir monolayers. (A) Estimated wavelength and **(B)** decay length of capillary waves on pure water covered with HP-BTBT-C*n* Langmuir monolayers (*n* = 10, blue dot; *n* = 14, green dot; *n* =18, orange curve) plotted as functions of surface area per molecule.

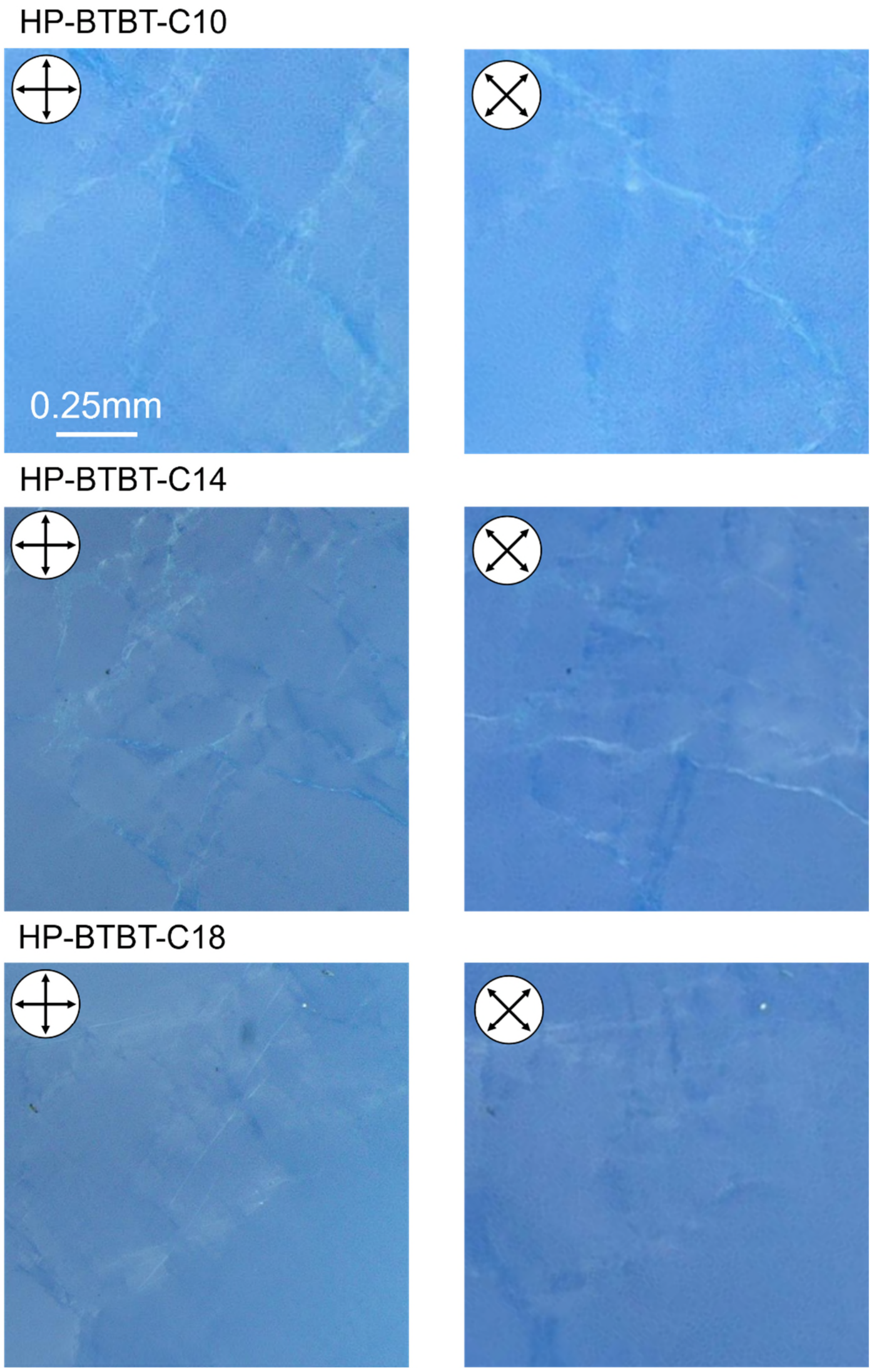

Fig. S8. Crystallinity and morphology of transferred Langmuir films. Crossed-Nicol polarized optical micrographs of HP-BTBT-C*n* (*n* = 10, 14, 18) Langmuir films transferred from the air−water interface onto glass substrates.

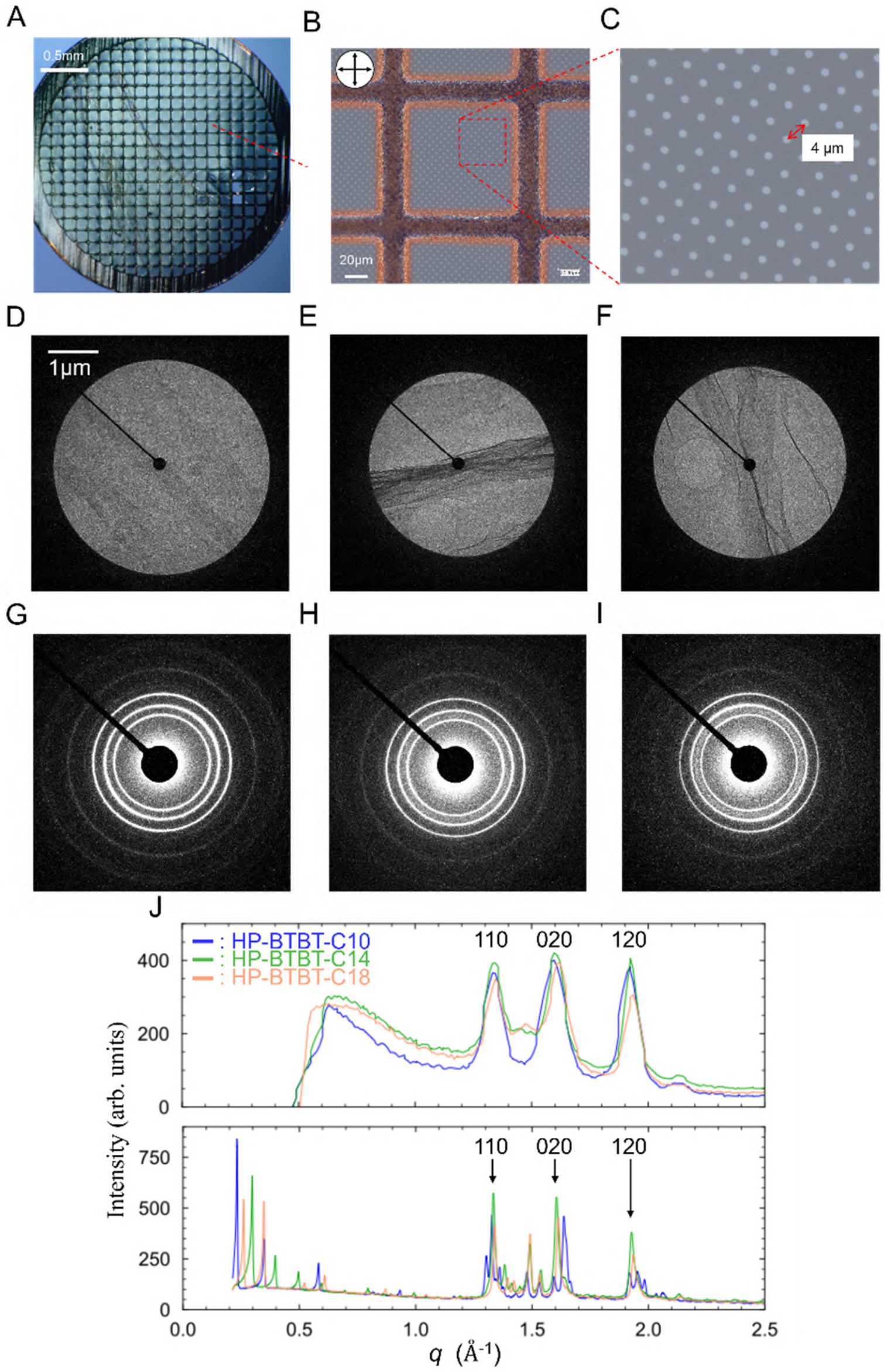

Fig. S9. Results of electron diffraction measurements for HP-BTBT-C*n*. (A) Crossed-Nichol polarized optical micrograph of an HP-BTBT-C10 film transferred onto a copper grid (opening size: 100 μm × 100 μm) coated with an ultrathin carbon film. **(B)** Magnified view of the copper grid coated with an ultrathin carbon film, showing periodically arranged holes with a diameter of 1 μm. **(C)** Further magnified view of the area indicated by the red dotted square in **(B)**. (**D−I**) Electron microscopy images (upper panels) and corresponding electron diffraction patterns (lower panels) of transferred monolayer films of **(D, G)** HP-BTBT-C10, **(E, H)** HP-BTBT-C14, and **(F, I)** HP-BTBT-C18. **(J)** Intensity profiles as a function of diffraction angle derived from the electron diffraction patterns of the transferred Langmuir films (top panel: n = 10, blue curve; n = 14, green curve; n = 18, orange curve). The curves are vertically offset for clarity. The bottom panel shows intensity profiles as a function of diffraction angle derived from powder X-ray diffraction measurements.

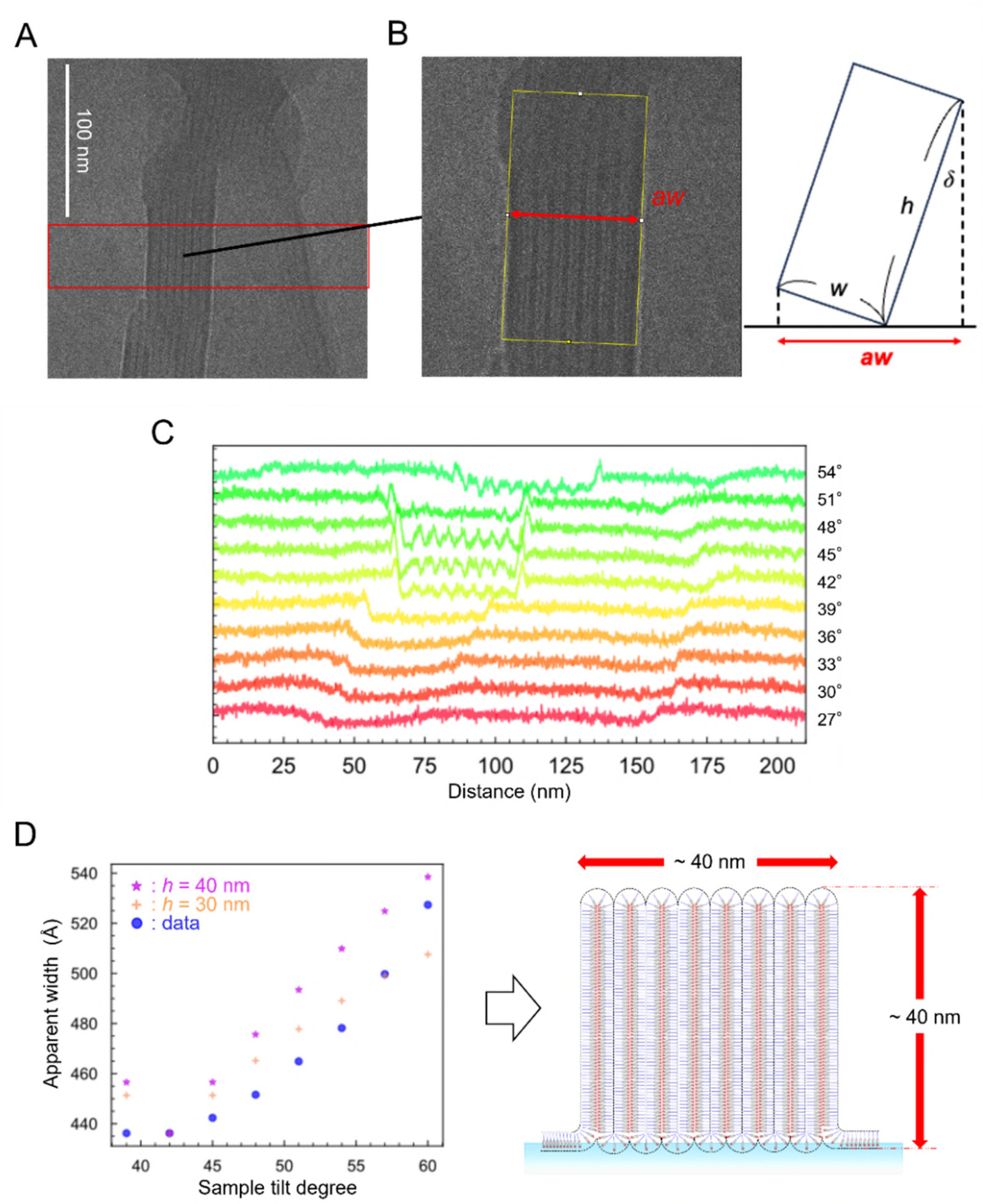

Fig. S10. Variation of intensity profiles in electron microscopy images and estimation of wrinkle height. (A) Representative electron microscopy image of a transferred monolayer film of HP-BTBT-C10, acquired at a sample rotation angle of 45°. **(B)** Schematic illustration of a simple rectangular wrinkle model used to estimate the true wrinkle height (*h*) and width (*w*) for a wrinkle tilted by an angle (δ), based on the apparent width (*aw*) observed in the electron microscopy images. **(C)** Intensity profiles extracted from electron microscopy images acquired at various sample rotation angles. The curves are vertically offset for clarity. **(D)** Plot of sample rotation angle versus apparent wrinkle width (*aw*, blue dots), derived from the experimental data shown in (C). Orange crosses and purple stars indicate simulated apparent widths for wrinkle heights of h = 30 nm and h = 40 nm, respectively. A schematic illustration of the proposed molecular wrinkle dimensions is also shown. This schematic does not represent directly resolved local molecular arrangements at the edges of multilayers.

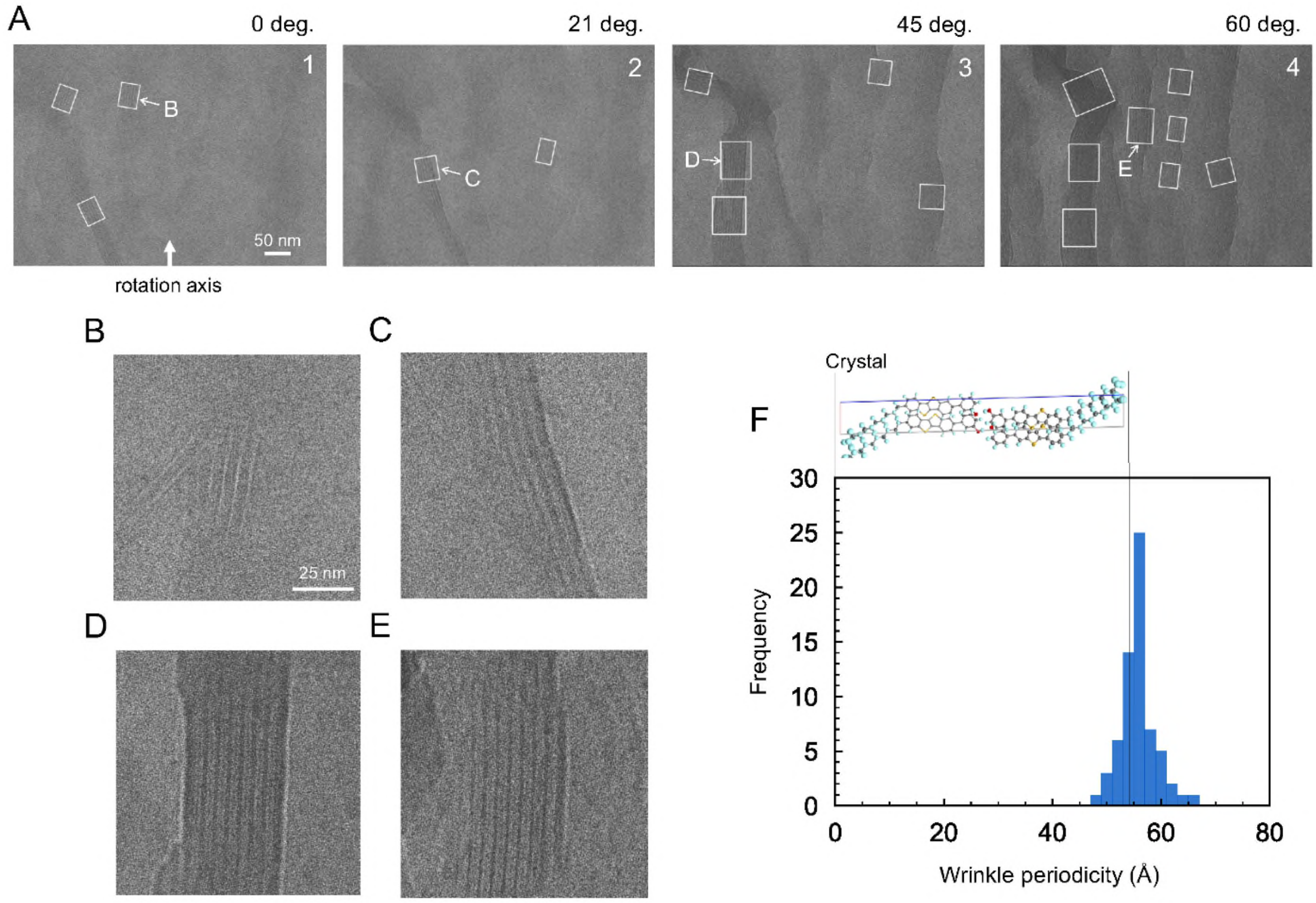

Fig. S11. Statistical analysis of stripe periodicity in wrinkle-like domains. (**A**) Tilt-series HRTEM images of a transferred HP-BTBT-C10 film shown in Fig. 3A. The wrinkle-like domains used for the analysis are enclosed by white rectangles. (**B −E**) Magnified views of the wrinkle-like domains enclosed by the white rectangle at tilt angles of 0° (B), at 21° (C), 45° (D), 60° (E). (**F**) Statistical analysis of the stripe periodicity in numerous wrinkle-like domains. The average periodicity is estimated to be 55Å, corresponding approximately to the thickness of a molecular bilayer. The wrinkle height and wrinkle area fraction are expected to increase with lateral compression and surface pressure, but they were not quantitatively controlled or systematically evaluated in the present study.

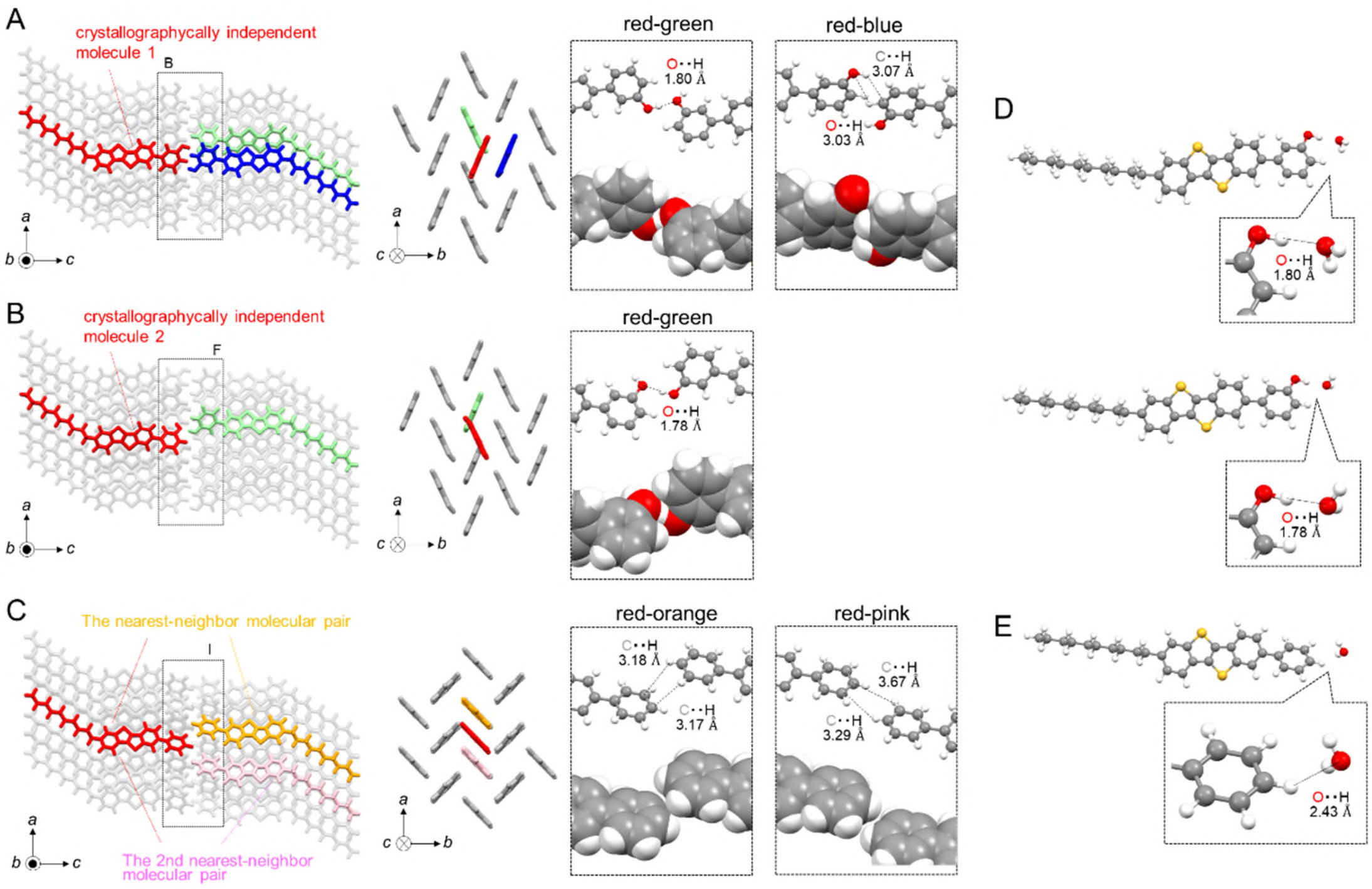

F

Materials	Combination	Energy (kcal/mol)
HP-BTBT-C10 (molecule 1)	red-green	−6.67
	red-blue	−4.91
	sum of red-gray pairs (11 pairs)	−5.01
	sum of total 13 pairs	−16.59
	with H_2O (Figure D upper)	−7.8
	with H_2O (Figure D lower)	−8.8
HP-BTBT-C10 (molecule 2)	red-green	−6.67
	sum of red-gray pairs (12 pairs)	−3.81
	sum of total 13 pairs	−10.48

G

Materials	Combination	Energy (kcal/mol)
Ph-BTBT-C10	red-orange (the nearest-neighbor)	−2.07
	red-pink (the 2nd nearest-neighbor)	−1.60
	sum of red-gray pairs (11pairs)	−3.49
	sum of total 13 pairs	−7.16
	with H_2O (Figure E)	−1.0

Fig. S12. Effect of the hydroxy group on intermolecular interaction energy. (**A, B**) Intermolecular configurations in the HP-BTBT-C10 crystal. Molecules exhibiting relatively strong interlayer intermolecular interactions relative to the reference molecule (red) are shown in blue and green, together with the corresponding interatomic distances. The two crystallographically independent molecules are shown in (A) and (B). (**C**) Intermolecular configurations in the Ph-BTBT-C10 crystal. Molecules exhibiting relatively strong interlayer intermolecular interactions relative to the reference molecule (red) are shown in orange and pink, together with the corresponding interatomic distances. (**D**) Locally stable configurations and interatomic distances for HP-BTBT-C10–water complexes and (**E**) those for Ph-BTBT-C10–water complex. (**F**) Calculated intermolecular interaction energies in the HP-BTBT-C10 crystal and for HP-BTBT-C10–water complexes. (**G**) Calculated intermolecular interaction energies in the Ph-BTBT-C10 crystal and for the Ph-BTBT-C10–water complex. Only the major intermolecular interactions extracted from the calculated results are shown. These results indicate that the hydroxy group enhances interlayer interactions in the HP-BTBT-C10 crystal and also strengthens interactions with water molecules.

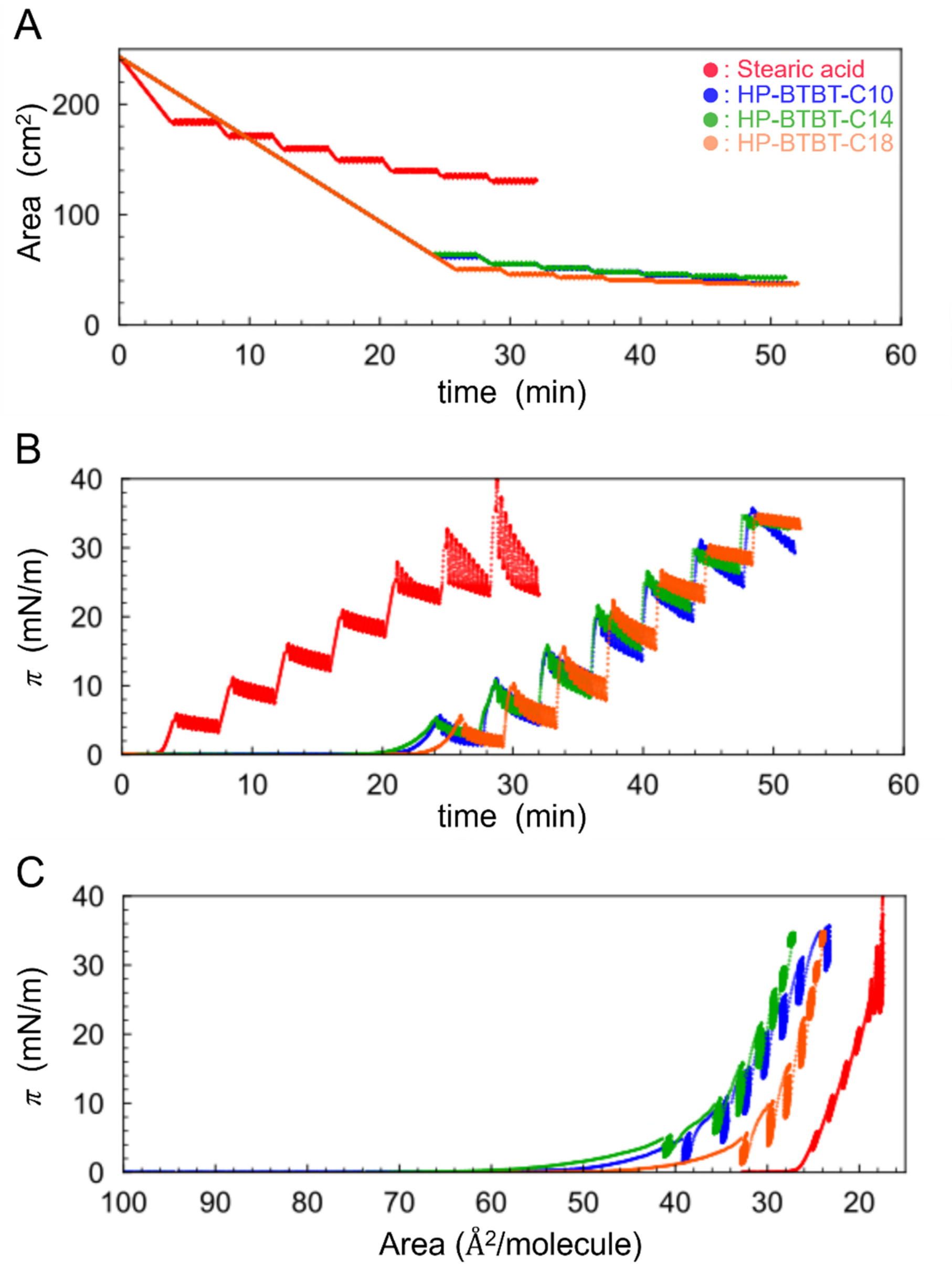

Fig. S13. Raw data for the barrier oscillation response of various Langmuir monolayers on water. **(A)** Surface area versus time (A−t) and **(B)** surface pressure versus time (π−t) curves for Langmuir monolayers of stearic acid (SA; red curve) and HP-BTBT-Cn (n = 10, blue curve; n = 14, green curve; and n = 18, orange curve), corresponding to the data used in Fig. 4 (A−D). For each measurement, the barrier was first compressed to reach the target surface pressure (initially set to 5 mN m^{-1}), followed by oscillation at a frequency of 50 mHz with a relative area modulation amplitude of 1%. After each oscillation sequence, the barrier was further compressed to increase the target surface pressure by 5 mN m^{-1}, and the oscillation experiment was repeated. **(C)** Reconstructed π−A isotherms obtained from the A−t and π−t data shown in (A) and (B).

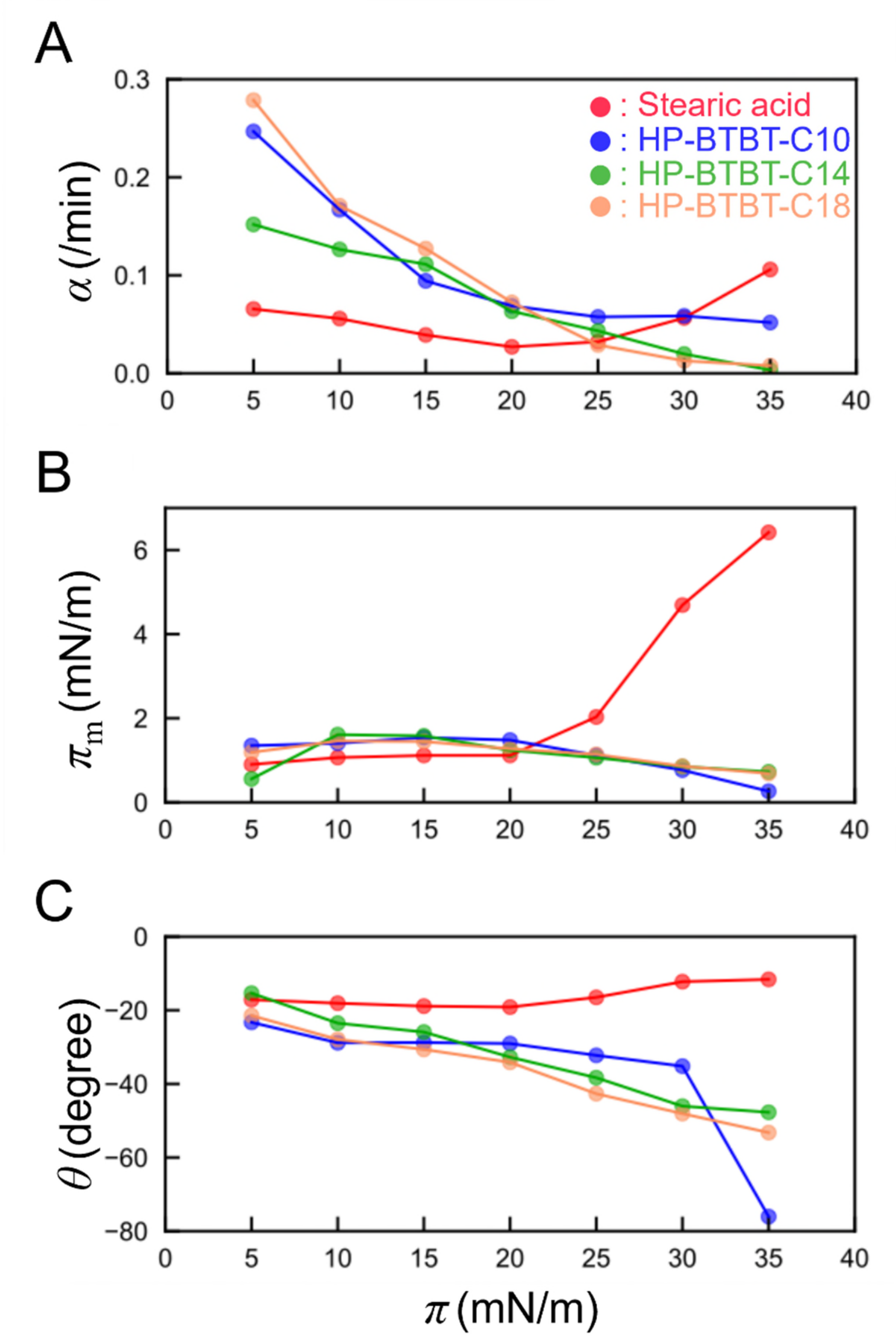

Fig. S14. Barrier oscillation response of various Langmuir monolayers on water. Results of barrier oscillation fitting for stearic acid (SA; red points) and HP-BTBT-C*n* Langmuir monolayers (n = 10, blue points; n =14, green points; and n =18, orange points). **(A)** Decay rate α, **(B)** oscillation amplitude π_m, and **(C)** phase retardation θ, obtained from the fitting analysis. The corresponding raw data are shown in Fig. S11.

List of Supplementary Movies.

Movie S1. Electron microscopy tilt series of self-folded HP-BTBT-C10 monolayer films. Tilt series images acquired from −60° to 60°. Selected frames are shown in Fig. 3A.

Movie S2. Electron microscopy tilt series of a wrinkle formed in an HP-BTBT-C10 monolayer. Tilt-series images correspond to the dataset shown in Fig. S10C.
